# Mitochondrial reprogramming of neutrophil extracellular traps in chronic obstructive pulmonary disease and asthma: toward a phenotype-driven therapeutic framework

**DOI:** 10.3389/fimmu.2026.1920684

**Published:** 2026-08-20

**Authors:** Pei Xu, Fen Xia

**Affiliations:** The Second Affiliated Hospital of Nanjing University of Traditional Chinese Medicine (Jiangsu Second Hospital of Traditional Chinese Medicine), Nanjing, Jiangsu, China

**Keywords:** chronic airway inflammation, corticosteroid resistance, mitochondrial reprogramming, neutrophil extracellular traps (NETs), phenotype-enriched trials

## Abstract

Chronic obstructive pulmonary disease (COPD), severe asthma, and asthma–COPD overlap (ACO) represent a major global burden of chronic airway inflammation, often remaining inadequately controlled by current corticosteroid and biologic therapies. Neutrophil extracellular traps (NETs) have emerged as a shared effector mechanism in these phenotypes, with neutrophil mitochondria acting as the upstream switch for sustained NET release and corticosteroid resistance. This review consolidates evidence that mitochondrial reprogramming of NETosis operates through four interlinked facets: mitochondrial reactive oxygen species (mtROS) production, the mitochondrial permeability transition pore, mitochondrial DNA release (mtDNA-DAMP), and PINK1/Parkin–PGC-1α–controlled mitophagy. These mechanisms drive a downstream spectrum including suicidal NETosis, vital mtDNA-NETosis, and gasdermin-mediated discharge. Disease-specific drivers (e.g., Nrf2–SLC7A11–GPX4 in COPD; IL-33/TSLP in asthma) reweight this common axis, resulting in distinct effector repertoires and steroid-response profiles. Therapeutically, the landscape is asymmetric: whilst NET-dissolution agents and type-2 biologics are approved, they address distal nodes. Conversely, mitochondrial-targeted antioxidants and metabolic regulators target the convergent root but remain in early development. We argue that translating this framework requires phenotype-enriched trials randomizing molecularly selected patients, coordinated bedside biomarker panels, and explicit inclusion of the ACO subgroup. A coherent framework, a set of candidate targets, and an explicit stratification logic are now defined, and the pivotal preclinical and early-phase clinical data needed to deliver such a programme are the necessary next step.

## Graphical abstract

**Figure:**
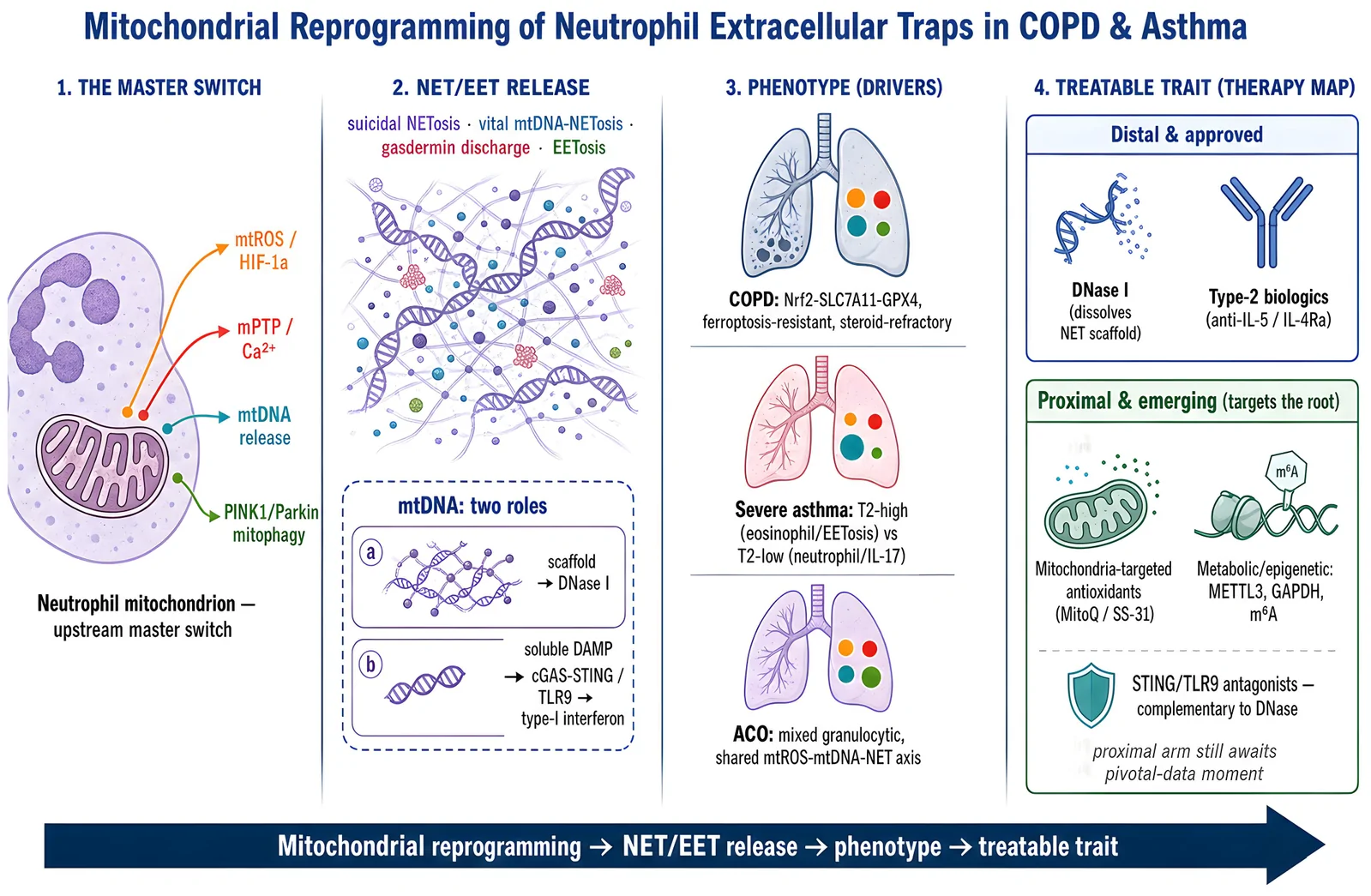

## 1 Introduction

Chronic obstructive pulmonary disease (COPD) and asthma together dominate the global burden of chronic airway inflammation. Global modelling estimated that 391.9 million adults aged 30–79 years had COPD in 2019, a population prevalence of 10.3% under the GOLD spirometric definition—with more than 80% of cases in low- and middle-income countries where age-standardised rates continue to rise alongside biomass exposure, occupational dust, and tobacco use (1). The 2024 GOLD update refined diagnosis, management, and prevention (2), reflecting recognition of COPD as a heterogeneous syndrome whose progression remains incompletely controlled by current pharmacotherapy. Severe difficult-to-treat asthma in adults consumes a disproportionate share of unscheduled care, oral corticosteroid exposure, and respiratory mortality despite representing a minority of asthma patients (3). The unmet need in both diseases, particularly type-2-low or corticosteroid-resistant phenotypes, provides the clinical impetus for revisiting cellular and molecular axes sustaining airway inflammation. Pooled meta-analytic data place the prevalence of asthma–COPD overlap (ACO) at ~2.0% in the general adult population but at 26.5% amongst asthma patients and 29.6% amongst COPD patients, indicating that overlap is the rule within either disease cohort (4). In a contemporary NHANES 2005–2018 analysis of 22,745 participants with mortality linkage through 2019, ACO conferred an all-cause mortality hazard of HR 1.91 (p < 0.001) versus participants without obstructive disease, with depressive symptoms mediating ~8% of excess risk (5). An independent 18-year Finnish cohort confirmed the same hierarchy (HR 1.29 for asthma, 1.50 for COPD, 1.26 for ACO) together with significant cardiovascular signals (6). The relevant clinical question is no longer whether COPD and asthma share inflammatory mechanisms but which mechanisms are shared in a mechanistically actionable way.

**Figure 1:**
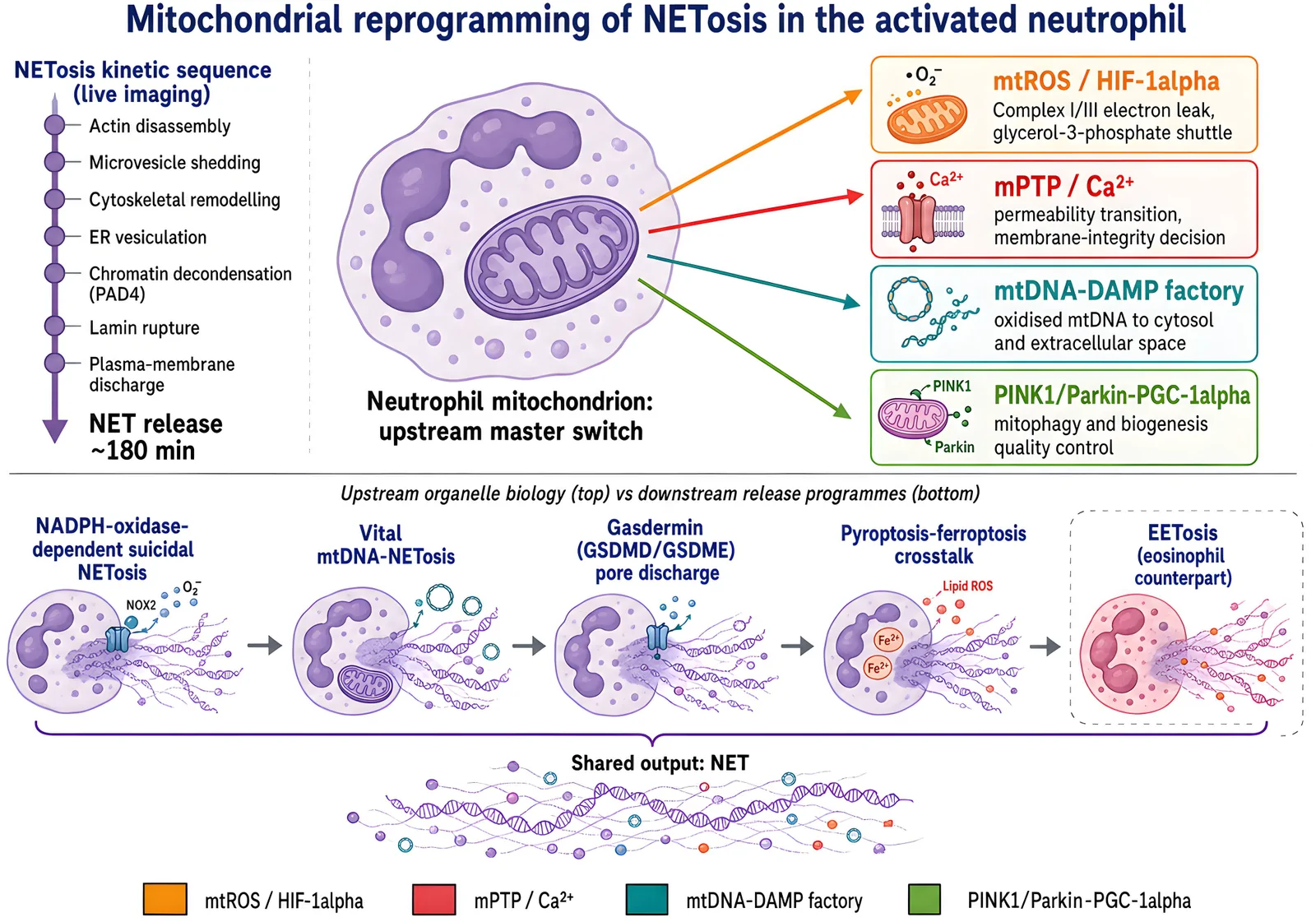
Mitochondrial reprogramming of NETosis in the activated neutrophil. Schematic overview of how the neutrophil mitochondrion licenses sustained NET release across cell-death modalities. The left timeline traces the canonical kinetic sequence resolved by live imaging, from actin disassembly through microvesicle shedding, cytoskeletal remodelling, endoplasmic-reticulum vesiculation, PAD4-dependent chromatin decondensation, and lamin rupture, to plasma-membrane discharge and NET release at approximately 180 min. The central element depicts the activated neutrophil and its mitochondrion as the convergent upstream organelle. The right-hand panel resolves the mitochondrion into four mechanistic facets that act together as an integrated upstream switch: an mtROS/HIF-1alpha module driven by complex I/III electron leak and the glycerol-3-phosphate shuttle, which licenses NET-permissive gene programmes; an mPTP–Ca2+ arbitrator of calcium and membrane-integrity decisions; an mtDNA-DAMP source exporting oxidised mtDNA into the cytosol and extracellular space; and a PINK1/Parkin–PGC-1alpha quality-control axis governing mitophagy and biogenesis. The lower band shows the downstream modality spectrum, spanning NADPH-oxidase-dependent suicidal NETosis, vital mtDNA-NETosis, gasdermin (GSDMD/GSDME) pore-mediated discharge, the pyroptosis–ferroptosis crosstalk branch, and EETosis, the eosinophil counterpart (previewed in Section 2.4). The horizontal divider separates the upstream organelle biology (Section 2.1) from the downstream release programmes (Section 2.2), and the colour key identifies the four mitochondrial facets. Bracketed numbers refer to citations in the main text.

**Figure 2:**
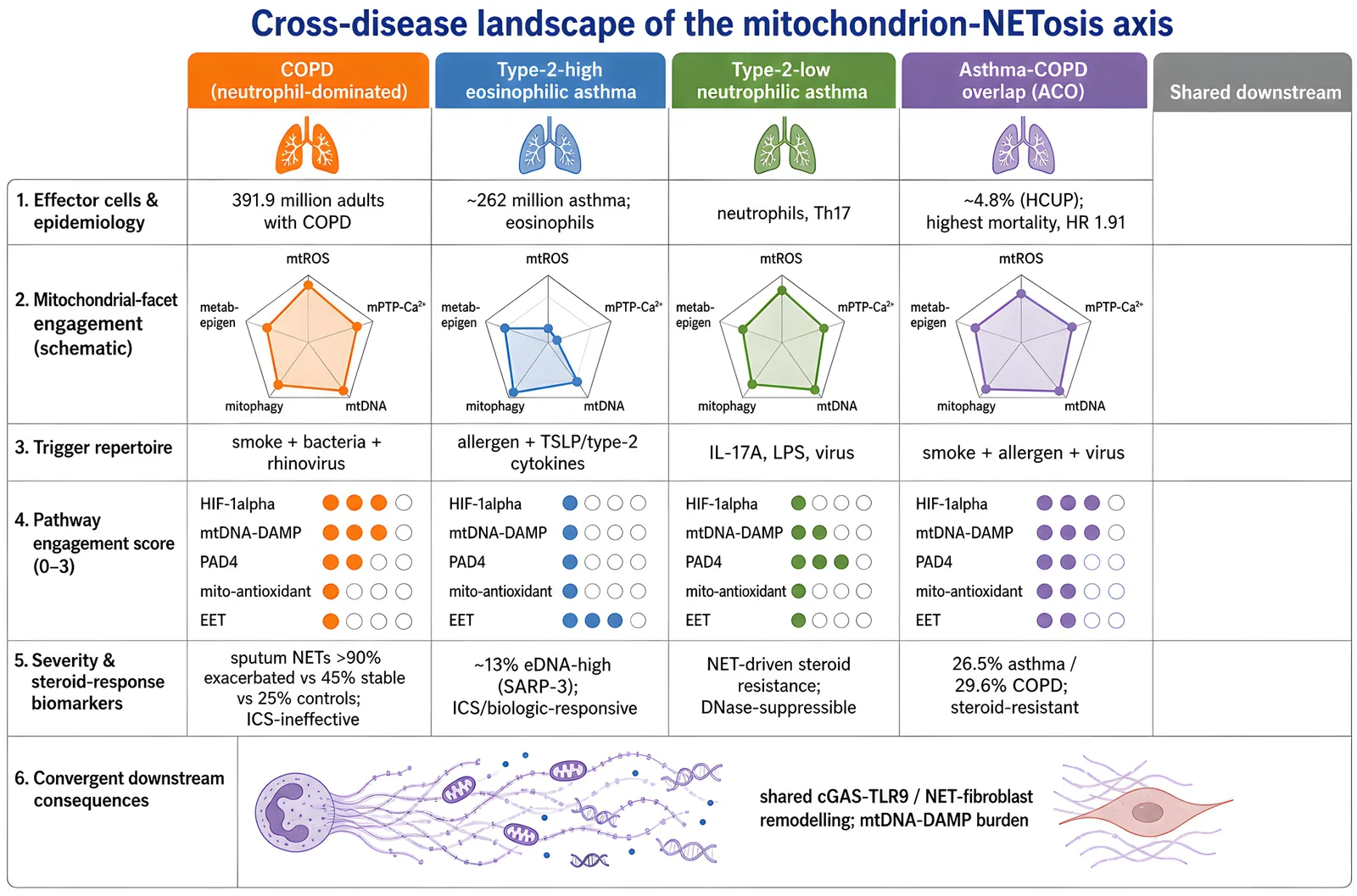
Cross-disease landscape of the mitochondrion–NETosis axis. Cross-disease matrix consolidating how a shared mitochondrion–NETosis framework is reweighted across COPD (neutrophil-dominated), type-2-high eosinophilic asthma, type-2-low neutrophilic asthma, and asthma–COPD overlap (ACO), alongside a shared-downstream column. The top row anchors each phenotype to its effector-cell repertoire and epidemiology, including global prevalence and excess-mortality estimates (391.9 million adults with COPD; approximately 262 million with asthma; approximately 4.8% ACO in the HCUP cohort; NHANES and Finnish multimorbidity hazard ratios). A second row shows relative mitochondrial-facet engagement across the five facets (mtROS, mPTP–Ca2+, mtDNA, mitophagy defect, and metabolic-epigenetic) as a schematic profile rather than measured coordinates. A trigger row summarises the dominant stimulus repertoire per phenotype (smoke and bacterial PAMPs; allergen and type-2 cytokines; LPS, virus, and IL-17A; mixed allergen-and-smoke loops in ACO). A pathway row indicates relative engagement across HIF-1alpha, mtDNA-DAMP, PAD4, mitochondrial antioxidants, and EET pathways as a graded, schematic strength scale rather than exact scores. A severity-and-steroid-response row anchors each phenotype to representative quantitative endpoints reported in the literature (sputum NET proportions, oral-corticosteroid reduction in the LIBERTY trials, and exacerbation rates). The final row collapses the convergent downstream consequences shared across phenotypes. Phenotype columns are colour-coded, and bracketed numbers refer to citations in the main text.

**Figure 3:**
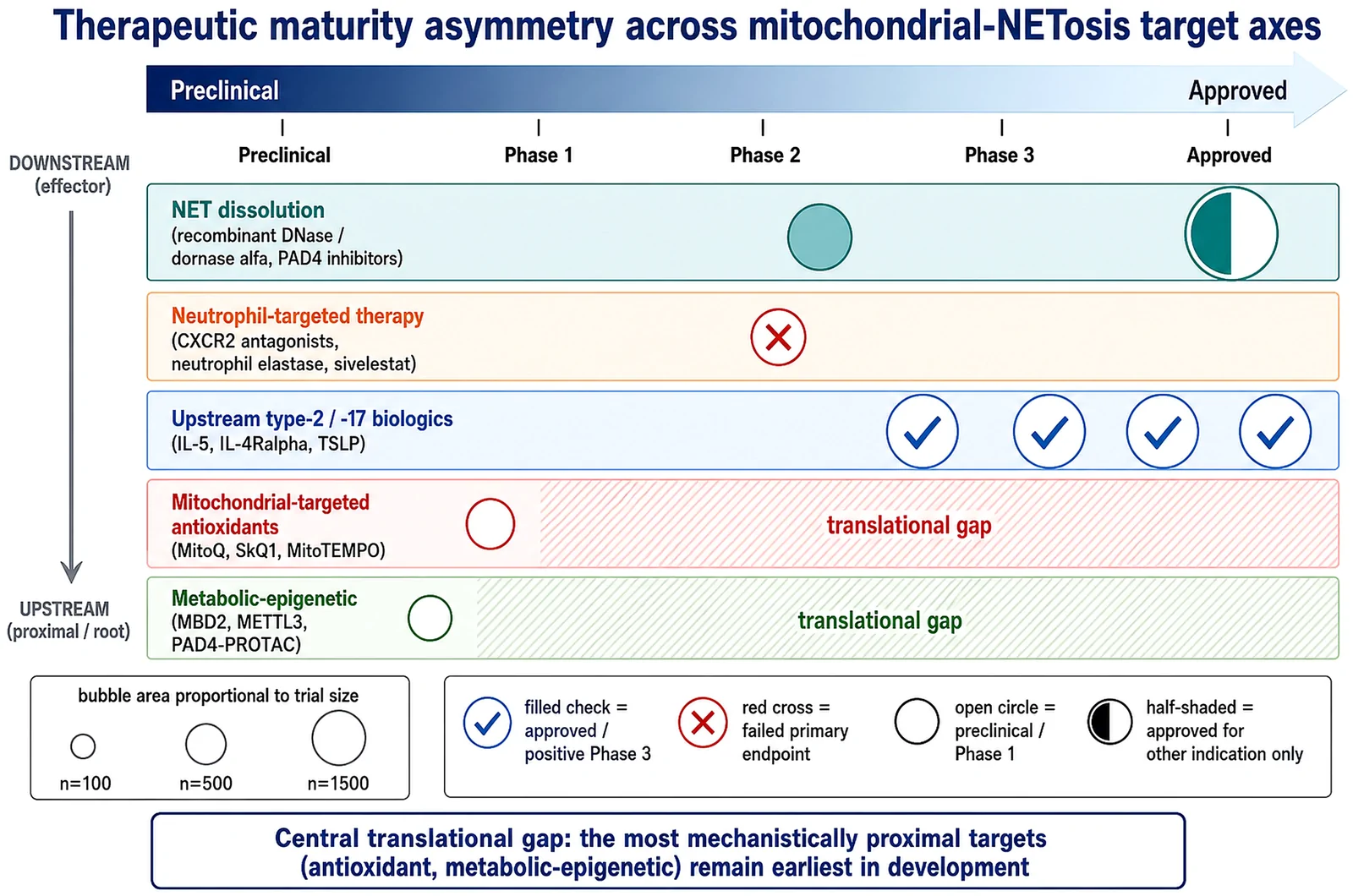
Therapeutic maturity asymmetry across mitochondrial–NETosis target axes. Pipeline-style visualisation of **Table 2**. Each horizontal ribbon represents one mechanistic target axis, and each bubble denotes a drug candidate placed at its current developmental stage (Preclinical, Phase 1, Phase 2, Phase 3, or Approved). Bubble size indicates the relative scale of the pivotal clinical evidence for each candidate and is intended as a visual guide rather than an exact measure. Five mechanistic ribbons are arranged from downstream to upstream: upstream type-2 biologics (IL-5, IL-4Ralpha, and TSLP); NET dissolution (recombinant DNase and PAD4 inhibitors); neutrophil-targeted therapy (CXCR2 antagonists and neutrophil-elastase strategies including sivelestat); mitochondrial-targeted antioxidants (MitoQ, SS-31, and SkQ1); and metabolic–epigenetic regulators (METTL3, GAPDH, and m6A-directed targets). Status glyphs inside each bubble encode trial outcome: a filled check marks an approved agent or a positive Phase 3 result; a red cross marks a failed primary endpoint; an open circle marks a preclinical or Phase 1 stage; and a half-shaded glyph marks partial efficacy or approval restricted to other indications. The two most mechanistically proximal ribbons, mitochondrial-targeted antioxidants and metabolic–epigenetic regulators, terminate at Phase 1 with hatched translational-gap bands marking the absence of completed later-phase development. The maturity gradient at the top runs from preclinical to approved, and ribbon colours match those used in **Figures 1** and **2**. The callout highlights the asymmetry that defines the central translational gap of the field. Bracketed numbers refer to citations in the main text.

Neutrophil extracellular traps (NETs), chromatin-protease structures released by activated neutrophils, originally described as antimicrobial defence (7), have emerged as one such candidate mechanism. Two decades of work establish that the same chromatin scaffolds and citrullinated histones that immobilise pathogens can, when released in excess or cleared inefficiently, sustain tissue inflammation and remodelling in COPD, severe asthma, and a wider spectrum of cross-organ disease (8, 9). The conceptual framing of airway inflammation has correspondingly shifted: The dominant paradigm of granulocyte-mediated inflammation has progressively incorporated mitochondrial dysfunction, programmed neutrophil cell-death modalities (apoptosis, pyroptosis, necroptosis, ferroptosis, and NETosis), and metabolic–epigenetic regulation through HIF-1α, GAPDH, and m6A methylation each providing distinct mechanistic entry points beyond the type 2 cytokine axis (10, 11). Mitochondrial biology has become the most provocative of these. Mitochondria in mature neutrophils, long dismissed as vestigial, retain a stable transmembrane potential and operate as plastic signalling hubs that licence NETosis through mtROS, supply mtDNA as a structural NET scaffold and downstream interferogenic DAMP, gate calcium and membrane-permeabilisation decisions, and turn over through PINK1/Parkin and PGC-1α-coordinated dynamics (12, 13). This review develops the argument that mitochondrial reprogramming of NETosis constitutes a unifying upstream switch across COPD, severe asthma, and ACO and that its individual nodes, mtROS source, NET assembly, NET-DNA scaffold, and NET-receptor signalling, define a tractable framework of treatable traits. To keep that argument navigable, the review carries three figures and two summary tables that recur in the text. **Figure 1** is a mechanistic schematic of how the mitochondrion licenses NET release, **Figure 2** sets the mechanism against the cross-disease landscape, and **Figure 3** maps each therapeutic strategy onto its stage of clinical maturity. **Table 1** consolidates the cross-disease mitochondria-NETosis and EETosis relationships, and **Table 2** consolidates the therapeutic strategies, so that the reader can move between the mechanistic narrative and its disease and treatment implications without losing the thread.

**Table 1:** Mitochondria–NETosis/EETosis axis in COPD, asthma, and asthma–COPD overlap.

Category	COPD	Asthma (T2-high/T2-low)	ACO
Dominant cell	Neutrophil; CD8^+^, macrophages (66, 104)	*T2-high*: eosinophil/CD4^+^/mast; *T2-low*: neutrophil/Th17 (76, 77, 79)	Neutrophil-dominant final common pathway (104)
Mitochondrial signature	↓ΔΨm, complex I/III/V deficits, mtROS; PINK1-mitophagy in smoke-exposed epithelium; respiratory-chain dysfunction in COPD neutrophils (12, 27, 71, 72)	Epithelial/smooth-muscle dysfunction; MitoQuinone rescues HDM eosinophilia (94, 95)	Same as COPD; T2D reprograms neutrophil mitochondria (48, 101)
Trigger	Cigarette smoke; rhinovirus and bacteria in exacerbation (67, 69, 72)	*T2-low*: IL-17A → CSF3; rhinovirus → IL-33 → PAD4-NETs; *T2-high*: TSLP/allergen → EETs with mtDNA release (80, 83, 84, 88, 89)	Smoke + allergen + virus + hyperglycaemic PAD4 priming (48, 101, 103)
Molecular axes	Nrf2-SLC7A11-GPX4 ferroptosis resistance; NCX1 Ca^2+^ entry; NE nuclear translocation (73, 74, 99)	*T2-low*: MBD2-JAK2-PAD4; CCL4L2; HIF-1/arginine; TNFAIP3/CXCR4/PADI4; *T2-high*: CCDC25-ILK-PKCα-CRTC1; autophagy-EETs (85, 86, 89, 90, 97, 98)	COPD ferroptosis + Th17-PAD4 convergence; IL-17-NETs/CXCL fibroblast crosstalk (72, 73, 80, 85, 100)
Downstream amplification	mtDNA → cGAS-TLR9 → NF-κB/IFN-I autoimmunity; elastase epithelial death; *cGas^-^/^-^*, *Tlr9^-^/^−^* protected (70, 72, 74)	EETs activate neuroendocrine cells via CCDC25; IL-17A-NETs drive pre-fibrotic fibroblasts; rhinovirus dsDNA correlates with type-2 (83, 89, 100)	Shared cGAS-TLR9 and NET-fibroblast remodelling (72, 100, 104)
Severity biomarker	NETs in >90% exacerbated vs. 45% stable vs. 25% smoking controls (p < 0.001); DNA-elastase tracks GOLD/FEV_1_/CAT (67, 68)	~13% of 399 SARP-3 patients “eDNA-high”; CLCA1-NAMPT AUC 0.903; TNFAIP3/CXCR4/PADI4 panel in 22%–60% ICS-poor severe asthma (82, 97, 98)	26.5% in asthma, 29.6% in COPD; 4.8% of 2.5 M hospitalisations; HR 1.91 mortality (4–6, 103)
Steroid response	ICS-ineffective; ferroptosis resistance sustains NETs despite dexamethasone; DNase I restores sensitivity (66, 73)	*T2-high*: ICS/biologic-responsive; *T2-low*: NET-driven steroid resistance; DNase I (not steroid) suppresses hyperreactivity (76, 77, 82, 85)	Shares steroid-resistant neutrophilic axis (104)

**Table 2:** Therapeutic strategies within the mitochondrion–NETosis axis: maturity asymmetry across five target groups.

Target axis	Mechanism of action	Representative agent (development stage)	Key supporting evidence	Principal limitations
NET dissolution	Cleave extracellular DNA scaffold; restore corticosteroid sensitivity by removing the chromatin-borne licensing signal	Inhaled rhDNase/dornase alfa (CF standard-of-care; severe COPD/asthma in Phases II–III)	CF pivotal trial: 28%-37% exacerbation reduction and FEV_1_ improvement of 5.6%-5.8% over 24 weeks (105); COVID-19 ARDS aerosolised rhDNase efficacy and safety (54–57)	Antibacterial defence narrowing; non-CF trial heterogeneity; mixed signal in eosinophilic phenotypes (60)
Mitochondrial-targeted antioxidants	Quench mtROS at the inner membrane; restrain NET-licensing redox cues; rescue mitophagy (99–105)	MitoQ, SkQ1, MitoTEMPO (preclinical/early Phase I)	COPD-derived ASM mitochondrial deficits MitoQ-reversible (90); MitoQ attenuates obese-allergic asthma (97); MitoTEMPO LPS-ALI rescue (98)	No completed COPD/asthma efficacy RCT; bioavailability, dosing, and lung-selective delivery unresolved (92–96)
Neutrophil-axis modulation	Block neutrophil recruitment, granule release, or NET programme	CXCR2 antagonists (Phase II); IL-8 axis blockers; PAD4 inhibitors (preclinical); NE inhibitors—sivelestat, alvelestat (47, 48)	Lazaar CXCR2 antagonist Phase II 5–50 mg BID failed dose-response over 26 weeks (77–83); Keir/Brightling biomarker-stratified rationale (51)	Off-target infection risk; lack of eDNA-enriched enrolment; phenotype-blind designs
Upstream type-2/-17 cytokines	Pre-empt eosinophil and neutrophil licensing upstream of NETosis	Mepolizumab, benralizumab, dupilumab (approved severe asthma); tezepelumab (anti-TSLP, broad)	MENSA: 47%-53% exacerbation reduction across baseline eosinophil strata (11–17, 130–135); NAVIGATOR pooled rate ratios 0.44 (T2-high) and 0.59 (T2-low) (84–89)	T2-low COPD/asthma only weakly responsive to anti-IL-5 axis; no convergent agent addressing all four mitochondrial faces
Metabolic-epigenetic	Erase NET-licensing epigenetic marks; restore mitophagy; degrade scaffold-only NET regulators	MBD2 inhibitors, METTL3 inhibitors, PROTAC degraders of PAD4 (all preclinical/discovery)	Peng: MBD2–JAK2–PAD4 axis active in HDM/OVA/LPS asthma models; Qu et al.: METTL3 inhibition reverses sepsis-ALI by restoring Sirt1-driven mitophagy (55)	No clinical trials; lung-selective delivery (LNP, sLNP) and off-target epigenetic toxicity unresolved

## 2 The non-energetic roles of mitochondria in neutrophils

The cross-disease puzzle motivating this review that type-2 cytokine biology alone cannot explain steroid-resistant phenotypes shared by COPD, severe asthma, and asthma–COPD overlap finds its candidate resolution in two underexamined elements: the cell whose biology has historically been considered the simplest, the neutrophil, and the organelle whose contribution has historically been judged minimal in that cell, the mitochondrion. Neutrophils were long treated as a metabolic outlier of the leukocyte family terminally differentiated cells deriving nearly all ATP from glycolysis and retaining only vestigial mitochondria. This portrait, codified in foundational reviews (14, 15), rested on abundance of glycolytic enzymes and granular ATP stores, the apparent dispensability of oxidative phosphorylation for the NOX2-driven respiratory burst, and rapid tissue apoptosis. A more recent reappraisal shows that mature neutrophils retain extensive mitochondrial networks with stable transmembrane potential, functioning as plastic signalling hubs whose contributions extend well beyond bioenergetics encompassing NET formation, chemotaxis, transcription, and cell-fate decisions, dynamically remodelled in response to extracellular cues (12, 16). The metabolic minimalism of the neutrophil is itself an active design feature rather than evidence of mitochondrial irrelevance. The mtROS signalling axis exemplifies this. Although neutrophil mitochondria contribute little to bulk ATP, they retain functional complexes I and III with measurable membrane potential and continue to leak superoxide and hydrogen peroxide as a low-level signal pool. This pool is regulated by the glycerol-3-phosphate (G3P) shuttle linking cytosolic glycolytic flux to a mitochondrial inner-membrane redox state, and mtROS produced through this shuttle is sufficient to stabilise hypoxia-inducible factor 1α (HIF-1α) in low-oxygen environments and to sustain HIF-1α-dependent neutrophil functions including chemotaxis, NET-licensing transcriptional programmes, and survival under inflammatory stress (13, 17, 18). The mitochondrial pyruvate carrier (MPC) constitutes a gateway between cytosolic pyruvate and the TCA cycle and shapes neutrophil immunometabolic behaviour (19), and its inhibition has been reported to bias neutrophils towards glycolysis and NETosis, with the integrated network glycolytic flux, mitochondrial respiration, and AMPK/mTOR/HIF-1α signalling operating as a coordinated immunometabolic system whose disruption biases neutrophil-effector functions towards NETosis (20, 21).

Mitochondrial membrane integrity provides a second decision node. The mitochondrial permeability transition pore (mPTP), formed by adenine nucleotide translocator and cyclophilin D under high Ca^2+^ and oxidative stress, drives loss of membrane potential, mtDNA leakage, and cytochrome-c release (22–24). IP3R-MAM-mitochondrial calcium signalling and the plasma-membrane Na^+^/Ca^2+^ exchanger 1 (NCX1) provide calcium-coupled redox cues that determine neutrophil-effector decisions, including NETotic versus survival programmes (25). Mitochondrial DNA can escape into the cytosol through three routes: mPTP opening, VDAC oligomers, and BAX/BAK-mediated permeabilisation of the outer membrane. This escape is a third regulatory axis for NETosis. Once in the cytosol, mtDNA is recognised by cGAS–STING and by TLR9, which trigger type-I interferon and NF-κB signalling. The same released mtDNA then plays two parts: a structural component of mtDNA-rich NETs and a paracrine danger signal for neighbouring cells (26–28). The molecular form of released mtDNA, intact circular molecules, fragments, and oxidised species determines downstream receptor engagement and inflammatory output (29). Mitochondrial quality control closes the regulatory loop. The PINK1-Parkin pathway selectively targets depolarised mitochondria for autophagic clearance, restraining propagation of mtDNA-driven inflammation and bounding the duration of any single NET-licensing event (30–32). PGC-1α coordinates the opposite arm of this circuit, driving NRF1/TFAM-dependent mitochondrial biogenesis to replenish capacity after acute damage; the balance of PINK1-Parkin clearance and PGC-1α-driven biogenesis defines a steady-state mitochondrial pool whose perturbation in chronic airway disease biases neutrophil cell-fate decisions towards sustained NETosis (33). The neutrophil mitochondrion can therefore be defined operationally as a four-faceted signalling node: an mtROS source linked to HIF-1α and glycolytic-flux control, an mPTP-gated arbitrator of calcium and membrane-integrity decisions, a factory for mtDNA-DAMP release into cytosol and extracellular space, and a substrate for PINK1/Parkin-mediated clearance and PGC-1α-driven biogenesis. This four-faceted view supplies the cellular grammar for the disease-specific syntheses that follow. Before developing that argument, two terms that recur throughout this review need to be distinguished, because we use them deliberately rather than as synonyms. Mitochondrial reprogramming refers to a regulated and in principle reversible remodelling of mitochondrial output, a shift in the balance of mtROS signalling, calcium handling, mtDNA release, and mitophagy that redirects neutrophil behaviour towards NET formation. Mitochondrial dysfunction refers instead to frank organelle failure, meaning loss of membrane potential, respiratory-chain deficits, and structural damage. The two states are linked but not identical, since reprogramming can precede and drive dysfunction whereas established dysfunction in turn deepens the reprogrammed state. Throughout the review, we reserve reprogramming for adaptive rewiring and dysfunction for pathological failure, and the distinction matters therapeutically because a reversible programme is a more plausible drug target than an organelle that has already failed.

## 3 Mitochondria as a bridge to NETosis across cell-death modalities

### 3.1 The canonical NADPH-oxidase-dependent NETosis programme

Granted that neutrophil mitochondria perform metabolic licensing, intracellular signalling, programmed-death gating, and intercellular communication, the next question is how this four-faceted role is actually wired into NETosis: which release modalities are mitochondria-dependent, and how that dependence interlocks with parallel programmed-cell-death systems. NETosis comprises a family of release modalities distinguished by kinetics, signalling, and neutrophil fate (34, 35). Mitochondria perform two non-redundant roles: generating mtROS and metabolite cues licensing chromatin decondensation, and supplying mtDNA extruded as NET scaffold and downstream interferogenic DAMP (36, 37). The canonical NOX-dependent route (Fuchs) couples NADPH-oxidase activation to nuclear delobulation, granule rupture, and plasma-membrane disruption absent in chronic granulomatous disease patients (38). Papayannopoulos mapped the downstream step: oxidase activation drives neutrophil elastase to the nucleus where it cleaves histones in synergy with myeloperoxidase, with elastase-knockout mice failing to form NETs in pulmonary *Klebsiella* infection (39). Raf-MEK-ERK signalling links upstream activation to oxidase mobilisation (40). PAD4 (peptidyl arginine deiminase 4) drives histone hypercitrullination and prevents nucleosomal compaction (41), with selective small-molecule PAD4 inhibitors that bind the calcium-deficient enzyme blocking both citrullination and NET formation in human and murine neutrophils (42). Thiam resolved the kinetic sequence by live imaging: actin disassembly, microvesicle shedding, microtubule/vimentin remodelling, ER vesiculation, chromatin decondensation, lamin rupture, and plasma-membrane discharge, with PAD4 required for decondensation and subsequent envelope events (43).

This four-faceted mitochondrial portrait must be situated within the physiological heterogeneity of the neutrophil compartment itself. Neutrophils are not a uniform circulating population: a substantial marginated pool resides within the pulmonary vasculature, where the dense capillary network and slow microvascular transit retain a reservoir of cells poised for immediate host defence (44, 45). Intravital imaging has shown that these marginated neutrophils crawl along the lung endothelium and phagocytose disseminated pathogens within minutes, establishing the pulmonary microvasculature as a functional immune niche rather than a passive conduit (45). Neutrophil kinetics are correspondingly rapid, with a circulating half-life of hours and continuous bone-marrow replenishment, so the cells sampled in sputum or bronchoalveolar lavage represent the recruited, activated fraction of a much larger and dynamically turning-over pool (46). Superimposed on this compartmentalisation is a maturational and activation continuum: single-cell transcriptomics resolves neutrophils along a conserved developmental spectrum rather than into discrete lineages (47), and ageing in circulation generates a proinflammatory CXCR4hi CD62Llo subset whose abundance is shaped by the microbiome and by chronic inflammation (48). Disease-specific subpopulations further refine this picture. In COPD airways, a PROK2-positive neutrophil subset dominates and exhibits elevated expression of matrix metalloproteases, NADPH-oxidase components, and NET-related genes, with serum PROK2 tracking neutrophilic inflammation and impaired lung function (49). In asthma, neutrophil-like low-density granulocytes expand with increasing severity and corticosteroid-refractory disease (50). In acute lung injury, transcriptionally distinct Fth1hi and Prok2hi neutrophil states drive sustained inflammation (51). We therefore emphasise that the mitochondrial reprogramming framework developed in this review applies most directly to the recruited, activated neutrophil subpopulations that dominate the chronically inflamed airway, rather than to the quiescent circulating or marginated reservoir; the mitochondrial state of the marginated pool, and the cues that licence its transition to a NET-competent phenotype, remain important boundary conditions for the model.

### 3.2 Shared executioners and vital mitochondrial NETosis

Membrane permeabilisation has emerged as a conserved exit step shared with pyroptosis and apoptosis. Sollberger showed that gasdermin D (GSDMD), the pore-forming executor of pyroptosis, is proteolytically activated by neutrophil proteases during NETosis in a feed-forward loop, with chemical GSDMD inhibition blocking both pyroptosis and NETosis (52). Zhu established a parallel apoptotic-to-NETotic route through gasdermin E (GSDME): mitochondrial and death-receptor apoptotic signalling promotes GSDME-dependent calcium mobilisation, H3 citrullination, nuclear DNA extrusion, and cytoplast formation, with GSDME loss preventing the disruption that completes NETosis (53). With the imaging data, pore activity converges as the permeabilisation event that turns a PAD4-primed neutrophil into a NET-releasing one, redefining the boundary amongst apoptosis, pyroptosis, and NETosis as one of shared executioners. In parallel, mitochondria support a distinct vital NETosis: GM-CSF priming followed by TLR4 or C5a-receptor stimulation drives viable human neutrophils to release NETs constructed predominantly of mitochondrial rather than nuclear DNA, with the responding cells surviving and remaining motile (37). *In vivo* imaging by Yipp captured live neutrophils releasing NETs during Gram-positive skin infection whilst preserving phagocytic capacity, producing anuclear cytoplasts requiring TLR2 and complement opsonisation (54). Lood characterised oxidised mtDNA-rich NETs as the dominant species elicited by lupus immune complexes, with extracellular oxidised mtDNA interferogenic through STING and mtROS scavenging reducing disease severity *in vivo* (36). Calcium-activated SK3 channel signalling, coupled to mtROS, drives rapid NETosis even when NADPH oxidase is genetically or pharmacologically disabled (55). Wong extended this to diabetic neutrophils, where elevated PAD4, *Padi4*-deficiency, and exogenous DNase I respectively licence, blunt, or accelerate NET-driven wound healing (56).

### 3.3 Released mtDNA as structural scaffold and soluble danger signal

Released mtDNA does not act only as structural scaffold. Liu and colleagues showed endogenous and especially oxidised mtDNA after tissue injury drives NETosis through cGAS-STING and TLR9, with increased elastase, extracellular DNA, ROS, and PAD4 expression (57). Mallavia demonstrated that cell-free mtDNA released during lung ischaemia–reperfusion triggers TLR9-dependent NETs in mice, whereas severe primary graft dysfunction in human lung transplant recipients tracks with high bronchoalveolar mtDNA and NETs together with relative DNase I deficiency (58). Aubé unified this framework: in sterile inflammation, TLR9, cGAS-STING, AIM2, and NLRP3 collectively detect cytosolic and extracellular DNA, with neutrophils expressing STING but not cGAS itself, supporting autocrine NETosis and paracrine activation of stromal and immune cells (59). The result is a self-amplifying loop architecture particularly relevant in non-resolving airway disease. A final layer recasts mitochondria as a quantitative gate rather than a binary switch. Li showed in severe COVID-19 that reduced GAPDH activity diverts flux to the pentose phosphate pathway and that pharmacological GAPDH inhibition is sufficient to induce NETosis through neutrophil-elastase activity and intracellular alkalinisation, a glycolytic checkpoint normally restraining spontaneous NETosis (60). Zhang described a NET–METTL3–m6A axis in sepsis-induced acute lung injury, with NETs activating m6A modification of HIF-1α and GPX4 to reprogram mitochondrial metabolism and drive alveolar ferroptosis (61, 62). Qu showed that the same NET–METTL3 axis impairs Sirt1-mediated autophagic flux in the alveolar epithelium (63). Sule placed mitochondrial dysfunction immediately upstream of NETosis in systemic lupus erythematosus, where the endoplasmic reticulum stress sensor IRE1α controls mtROS generation and caspase-2 activation, and IRE1α inhibition reduces mtROS, NETosis, and autoantibody formation (64). Pérez-Figueroa placed NETosis within a continuum of regulated neutrophil deaths, apoptosis, autophagy, pyroptosis, necroptosis, NETosis, and necrosis, each modulated by mitochondrial ROS, membrane potential, calcium, and metabolic state (65). The integrative view from recent reviews is that mitochondria do not merely permit NETosis but quantitatively determine which release modality occurs, on what timescale, and with what downstream interferogenic capacity (66, 67). Katsoulis-Dimitriou showed that rhinovirus-driven NETosis is amplified in COPD and correlates with exacerbation severity, with NETosis inhibition or DNase I protective in a virus-exacerbated COPD model (68), and Zeng found platelet-derived 5-HIAA elevated in COPD lung tissue and promoting aryl-hydrocarbon-receptor-dependent NET formation in cigarette-smoke/LPS-treated mice (69). The continuing accumulation of NETs as a source of citrullinated self-antigens, complement activators, and bystander activators of B and T cells argues that the mitochondrion–NETosis axis is a core-modifiable node of chronic airway inflammation rather than a niche cell-biological detail (70). Because the same molecule is being asked to do two very different jobs, it helps to separate them, since they differ in where they act, how fast they act, which receptor they engage, and which drug can reach them. As a structural element, extruded mtDNA becomes part of the extracellular chromatin lattice of the NET itself, physically interwoven with nuclear DNA, histones, and granule proteins. In that guise, it acts in the extracellular space over the minutes-to-hours timescale of NET assembly and persistence, and the therapeutic lever against it is enzymatic degradation, since inhaled recombinant DNase I dissolves the scaffold whichever nucleic acid seeded it. As a soluble danger signal, the same DNA behaves quite differently. Fragments that reach the cytosol are sensed by cGAS–STING, released or endosomal species engage TLR9, and oxidised fragments generated by base-excision repair exit through mPTP- and VDAC-dependent channels to activate the NLRP3 inflammasome and type-I interferon signalling (71, 72). This danger-signal role plays out on a signalling timescale, is specified by receptor rather than by mass, and is addressed not by DNase but by pathway inhibitors such as STING, TLR9, or cGAS antagonists that leave the extracellular scaffold intact. What couples the two roles is oxidation, because oxidised mtDNA both resists nuclease degradation, which prolongs its life as a scaffold, and is the most interferogenic ligand for the cytosolic sensors, so a single population of oxidised molecules can serve as scaffold and danger signal at once rather than as two separate pools (73). That dual identity carries a direct clinical corollary, namely, that DNase I and STING or TLR9-directed agents are not interchangeable but complementary, the former clearing deposited chromatin and the latter interrupting the self-amplifying interferon loop that sustains it, a therapeutic pairing we return to in Section 2.6 and **Figure 3**. The modality spectrum, NOX-dependent suicidal NETosis, NOX-independent vital mtDNA-NETosis, EETosis, ferroptosis, and pyroptosis, converges on shared mitochondrial gating points (mPTP, GSDMD/GSDME, PAD4-citrullination), distinguishing the canonical infection-driven programme from the steroid-resistant, mtDNA-rich phenotypes that dominate chronic airway disease. The neutrophil-dominated COPD phenotype provides the cleanest setting in which to test this framework (**Figure 1**).

## 4 NETs and mitochondrial dysfunction in COPD

COPD is the indication where the mitochondrion-NETosis cycle is most directly evidenced histologically, biochemically, and at the level of mitochondrial-dynamics protein abundance, and where neutrophil dominance brings the framework developed above into sharpest focus. Hogg’s morphometric study (159 patients, GOLD 0-4) established that COPD progression associates with small-airway-wall thickening, luminal mucous exudates, and infiltration by neutrophils, macrophages, lymphocytes, B cells, and lymphoid follicles, with neutrophil infiltration amongst the strongest stage-advancement correlates (74). The framing of small conducting airways <2 mm as a “silent zone” integrates histopathology, mechanics, and molecular evidence supporting small-airway disease as the principal obstruction site (75). Christenson’s seminar situates this within a life-course view incorporating omics, imaging, biomarker-driven subtyping, and recognition that COPD risk extends beyond tobacco to early-life factors (76). Brightling and Greening distinguish neutrophil-associated COPD (NLRP3, type-1/type-17) from minority eosinophilic type-2 and autoimmune phenotypes: inhaled corticosteroids are effective in eosinophilic inflammation, anti-IL-5 biologics have disappointed in COPD, and broad neutrophil suppression risks infection (77). Direct NET evidence in the COPD airway came from Grabcanovic-Musija, who detected NETs by confocal and electron microscopy in >90% of sputum samples from exacerbated COPD versus 45% of stable COPD, 25% of smoking controls, and <5% of non-smokers (p < 0.001), with quantitative NET area correlating with airflow limitation (78). Dicker quantified sputum DNA-elastase and histone-elastase complexes in serial samples: NET complexes correlated with the composite GOLD severity score (p < 0.0001), FEV_1_, and COPD assessment test symptoms and were elevated in frequent exacerbators, establishing NET concentration as a continuous severity biomarker (79). The mechanistic synthesis by Uddin frames “unresolving neutrophilia” with NET formation as a NETopathy phenotype in refractory neutrophilic lung disease, amplified by *Staphylococcus aureus*, rhinovirus, and *Aspergillus* (80). Trivedi et al. situate these observations alongside experimental evidence that NETs directly induce epithelial and endothelial death and impair pulmonary function, framing NET burden as both marker and driver of COPD progression (81).

Wiegman placed mitochondrial dysfunction upstream, who showed in ozone-exposed mice a tractable oxidative-stress model that lung inflammation and airway hyperresponsiveness associate with decreased mitochondrial membrane potential, increased mitochondrial oxidative stress, and reduced expression of complexes I, III, and V, with the mitochondria-targeted antioxidant MitoQ reversing both lesions; equivalent mitochondrial abnormalities and MitoQ-responsive inflammation occurred in airway smooth-muscle cells from COPD patients (82). This connects to the §2.1 landscape, loss of membrane potential, complex deficits, and mtROS amplification, and to evidence that cigarette-smoke exposure drives PINK1-dependent mitophagy to a necroptotic outcome in airway epithelium, with *Pink1*-deficient mice protected from smoke-induced airspace enlargement and mucociliary disruption. A mechanistic bridge between mitochondrial dysfunction and NET-driven inflammation has emerged from three studies. Chen demonstrated that COPD-patient neutrophils released greater NET quantities in response to cigarette-smoke extract than control cells, that mitochondrial respiratory-chain dysfunction drove this, and that oxidatively damaged NETs drove airway-epithelial proliferation, NF-κB activation, type-I interferon production, and dendritic-cell maturation through cGAS and TLR9 signalling; *cGas^-^/^-^* and *Tlr9^−^/^−^* mice were protected in long-term cigarette-smoke COPD, and NETs-DNA blockade was protective (83). Wang identified a parallel mechanism explaining the steroid-resistant nature of severe COPD: extensive NET formation in heavy long-term smokers was tightly linked to glucocorticoid resistance, with cigarette-smoke extract conferring ferroptosis resistance via the Nrf2–SLC7A11–GPX4 axis to sustain NET production despite dexamethasone exposure, and DNase I administration restoring glucocorticoid sensitivity in COPD-model mice (84). A separate Wang study identified a tractable upstream target: the selective neutrophil-elastase inhibitor GW311616A prevented cigarette-smoke-induced NET formation by blocking elastase nuclear translocation and chromatin decondensation, reduced ROS production and migration defects in human neutrophils, and substantially attenuated airway leukocyte infiltration, goblet-cell hyperplasia, and emphysema-like alveolar destruction in a chronic smoke mouse model (85). The review by Keir and Chalmers consolidate these findings across COPD, severe asthma, cystic fibrosis, and bronchiectasis, framing NET burden as a unifying severity mechanism whilst emphasising the therapeutic challenge of suppressing NETosis without disabling antimicrobial defence (86). The COPD literature now defines a coherent axis: cigarette smoke and oxidant exposure drive mitochondrial dysfunction in airway cells and infiltrating neutrophils, mitochondrial damage amplifies NETosis through mtROS, oxidised mtDNA, and ferroptosis-resistant survival, NETs then sustain inflammation through cGAS-TLR9 signalling on bystander epithelium, and the resulting cycle resists conventional corticosteroid therapy. The same body of work appears at moments to make ferroptosis both a friend and an enemy of the neutrophil and that apparent contradiction resolves once cell type and context are specified. In the steroid-resistant COPD neutrophil, cigarette-smoke-driven engagement of the Nrf2–SLC7A11–GPX4 axis confers ferroptosis resistance, so the neutrophil survives oxidative stress and keeps releasing NETs despite dexamethasone (85). In sepsis-associated acute lung injury, the picture inverts, because NET-activated m6A modification of GPX4 now drives ferroptosis in the alveolar epithelial cell, a different cell that sits downstream of the NET rather than within the neutrophil (61, 62). Ferroptosis is therefore protective for the effector neutrophil yet destructive for the bystander epithelium, and its direction of effect turns entirely on which cell is under consideration.

## 5 Asthma: phenotype-specific engagement of the mitochondrion–NETosis axis

Whereas COPD inflammation is neutrophil-dominated, asthma is heterogeneous across phenotypes in dominant effector cell, cytokine environment, and corticosteroid response. Wenzel et al. recast asthma as phenotypes refined into endotypes linking biology to phenotype through statistical clustering (87), partitioning into type-2-high disease (eosinophilic, IL-4/IL-5/IL-13-driven, responsive to corticosteroids and type-2 biologics) and type-2-low disease (neutrophil-predominant or paucigranulocytic, low type-2 cytokines, and poor corticosteroid response) (88). Innate-immune machinery bridges these compartments through IL-1β signalling (89). The mitochondrion-NETosis axis (§2.2) engages each endotype through distinct molecular cues. Type-2-low neutrophilic asthma is the principal unmet need (90), linked to a Th17 program where IL-17A and IL-17F drive inflammation, smooth-muscle remodelling, and hyperresponsiveness. Ouyang showed through RNA sequencing that IL-17A and dexamethasone co-induce CSF3 in type-17 neutrophilic models, with dexamethasone alone exacerbating disease and anti-IL-17A, cyanidin-3-glucoside, or CSF3 depletion restoring corticosteroid responsiveness (91). Lipocalin-2 and serum amyloid A are type-17 serum biomarkers, IL-17A-correlated, corticosteroid-resistant, and co-induced through CEBPB (92). Nabe positioned NETosis as a proximal effector of corticosteroid-resistant asthma, congruent with IL-17A-driven chronicity. Lachowicz-Scroggins’s study established the clinical NET-severity link: sputum eDNA in 399 patients from SARP-3 and 94 controls identified ~13% as “eDNA-high” with neutrophil/inflammasome activation and DNase-preventable epithelial injury (93). Toussaint defined the viral-NET link in exacerbation: rhinovirus-induced host dsDNA release correlated with type-2 allergic inflammation, with elastase inhibitor or DNase blockade protecting mice (94). The proximal trigger was mapped to IL-33: rhinovirus-given chronic-asthma mice showed an early neutrophil-NETotic phase (day 1) followed by a type 2 phase (days 3-7), both ablated by HpARI (an IL-33-blocking protein), with exogenous IL-33 recapitulating PAD4-dependent airway NETosis (95). Tsai identified through transcriptomes of Taiwanese controlled/uncontrolled-asthma children a neutrophil-immunity module distinguishing uncontrolled disease and a NET-abundance corticosteroid-non-response correlate; CCL4L2 was the strongest neutrophil-specific corticosteroid-failure transcript, with DNase I—not corticosteroid—inhibiting airway hyperreactivity in neutrophilic mice (96). The MBD2–JAK2–PAD4 epigenetic-to-NETosis axis connects NET programming to methylation-dependent gene control, MBD2 acting upstream of JAK2 and PAD4 (97). NET burden in asthma is a continuous severity biomarker, corticosteroid-non-response predictor, and disruption-restorable target (98).

Eosinophils generate a parallel mechanism, eosinophil extracellular traps (EETs), in type-2-high asthma. Yousefi first showed LPS-stimulated IL-5- or IFN-γ-primed eosinophil release mtDNA via an ROS-dependent process within 1 s, independent of cell death, with antimicrobial activity in caecal-ligation-puncture sepsis (99). Lu showed that bronchoalveolar EETs associate with asthma severity; augment goblet-cell hyperplasia, mucus, inflammation, and type-2 cytokines; are triggered by allergens via thymic stromal lymphopoietin; and activate pulmonary neuroendocrine cells through CCDC25–ILK–PKCα–CRTC1 (potentiated by eosinophil peroxidase) to amplify allergic inflammation; CCDC25 inhibition alleviated this *in vivo* (100). Silveira established EET autophagy dependence: 3-methyladenine reduced eosinophil number, peroxidase activity, goblet-cell hyperplasia, NF-κB activation, oxidative stress, mitochondrial-energy defects, and EETs in ovalbumin asthma (101). Germic showed temporal dissociation: complement-C5a stimulation of primed eosinophils produced maximal degranulation within 1 min, with bulk mtDNA release proceeding much longer—reframing EETs as a sequential programme (102). Fukuchi and Shen consolidated EETosis visualisation and noted that classical Charcot–Leyden crystals closely associate with EETosis (103, 104). EETosis is thus the type-2-high counterpart of the NETopathy phenotype dominating type-2-low neutrophilic asthma and COPD; both converge on a mitochondrion-derived DNA-DAMP whose downstream pathology overlaps but whose cellular origin differs by endotype. Koranteng synthesised evidence that mitochondrial dysfunction and altered metabolism occur in airway epithelial and smooth-muscle cells from asthma patients, with severe eosinophilic asthma associated with peripheral and lung eosinophilia and treatment-resistant exacerbations (105). Chandrasekaran provided direct mtROS-target evidence: in a house-dust-mite obese-allergic mouse model, the mitochondria-targeted antioxidant MitoQuinone reduced eotaxin, eosinophilia, hyperresponsiveness, and remodelling in both lean and obese mice (106). Savin reviewed lung fibrosis in fatal and long-term asthma as irreversible inflammation/remodelling consequence (107). The mitochondrial-respiratory-chain dysfunction, mtROS amplification, ferroptosis-resistant neutrophil survival, and ER-stress cascades from §2.1 and §2.2 have asthma counterparts whose effector readout reflects the dominant cellular substrate: NETs in the neutrophilic endotype, EETs in the eosinophilic endotype, combined remodelling, and fibrotic outcomes in severe disease. The synthesis is generating practical biomarkers. Han identified through bioinformatic and machine-learning approaches TNFAIP3, CXCR4, and PADI4 as NET-associated severe-asthma biomarkers, motivated by ~22%-60% of asthma patients remaining poorly controlled despite inhaled corticosteroids, with CXCR4 and PADI4 promoting NETs and TNFAIP3 inhibiting them (108). Liu profiled sputum from 53 asthma patients (27 eosinophilic, 20 neutrophilic) and 53 controls through integrated proteomics, metabolomics, WGCNA, CIBERSORT, and machine learning: eosinophilic asthma showed Th2 signalling with dysregulated histidine/purine metabolism and ROS-and-cytokine production, and neutrophilic asthma showed neutrophil oxidative stress, HIF-1 activation, arginine-metabolism disturbance, hypoxia response, and NF-κB enrichment, with a CLCA1–NAMPT two-biomarker model discriminating endotypes at AUC 0.903 (109). Liao identified Na^+^/Ca^2+^ exchanger 1 (NCX1) as a calcium gate upregulated in neutrophils from chronic–bronchitis–emphysema: cigarette smoke activates NCX1 reverse-mode transport to raise intracellular calcium and amplify NETs, with neutrophil-specific *Slc8a1* deletion or NCX1 inhibition suppressing NETs and ameliorating emphysematous changes in mice (110), a target potentially applicable to neutrophilic asthma. Cai integrates these signals into neutrophil-fibroblast crosstalk, with fibroblasts secreting CXCL1, CXCL8, and CXCL12 to recruit neutrophils that release NETs, elastase, and cytokines to modulate fibroblast function, with IL-36 in COPD versus IL-17-NETs in pulmonary fibrosis defining tissue-specific risk (111). Ganesh used integrated human/mouse sepsis models to show that type-2-diabetes reprograms neutrophil mitochondria defining the immunometabolic axis constraining phagocytosis-versus-NETosis in diabetic neutrophils (112). Li described a site-specific low-dose nanotherapy delivering immunoregulatory cargo for asthma to inhibit neutrophilic airway inflammation locally (113). The asthma synthesis converges with the COPD synthesis on a single mtROS–mtDNA–NET axis that is reweighted, not replaced, across endotypes: eosinophil licensing layered on top in T2-high disease, and a steroid-resistant neutrophilic NETopathy dominating T2-low and severe phenotypes. When these two endotype-distinct mitochondrial programmes co-occur as in roughly one quarter of adults with chronic airway disease, the convergent phenotype is asthma–COPD overlap. This rapid, vital release contrasts with the delayed, often suicidal NETosis programme in neutrophils, underscoring that the mitochondrion–DNA–DAMP axis operates on distinct timescales and cell-fate contexts across granulocyte subtypes, even whilst sharing the same downstream effector molecules.

## 6 The asthma–COPD overlap and a unifying view of mitochondrial NETopathy

The clinical importance of overlap was established in the Introduction (Section 1): A nationally weighted analysis of the United States Healthcare Cost and Utilization Project Nationwide Readmissions Database (2012–2015), examining 2,522,013 hospitalised patients of whom 4.8% had ACO, found ACO patients younger than COPD-alone patients (mean age 63 vs. 69, p < 0.05) and at higher rate of respiratory-failure-related hospitalisation, identifying ACO as a high-burden subgroup warranting targeted study rather than dilution into either parent diagnosis (114). The biological rationale for treating ACO as distinct rather than as co-occurrence was articulated by Aoshiba and Nagai: in asthma, the infiltrate is dominated by CD4 T lymphocytes, eosinophils, and mast cells with epithelial injury and reticular basement-membrane thickening; in COPD, CD8 T lymphocytes and macrophages predominate with alveolar-wall destruction; and in severe forms of both diseases, neutrophil infiltration becomes prominent, identifying a final common pathway in which neutrophil-driven, mitochondrion-amplified NETopathy converges across endotypes (115). The same mitochondrial dysfunction driving NET production in COPD neutrophils through ferroptosis resistance and cGAS-TLR9 signalling operates in severe neutrophilic asthma through Th17-driven inflammation and produces EETs in eosinophilic asthma through viable-cell mitochondrial DNA release. The unmet need, corticosteroid-resistant inflammation in patients with progressive lung-function decline, is largely the same whether the index diagnosis is COPD, severe neutrophilic asthma, or ACO. The mitochondrion–NETosis axis therefore does not partition the diseases but identifies a shared mechanism whose therapeutic exploitation will require phenotyping by molecular and cellular signatures rather than by legacy diagnostic categories. Systemic comorbidities further shape this axis: type 2 diabetes, present in ~30% of severe COPD patients and increasingly recognised as an asthma-severity amplifier, primes neutrophils for excessive NETs through hyperglycaemia-driven PAD4 upregulation and reprograms mitochondrial function to elevate NETosis under sepsis (112). Fibrosis as a long-term endpoint of severe asthma and emphysematous COPD has been mechanistically linked to neutrophil-fibroblast crosstalk through CXCL chemokines and IL-17-carrying NETs (111). These cross-organ links argue that NET-targeted strategies for the airway require evaluation for systemic effects in multi-morbid high-mortality patients. These cross-disease parallels and the disease-specific upstream drivers are consolidated in **Figure 2** and **Table 1**.

It should be acknowledged that the four strata used throughout this review, COPD, type-2-high asthma, type-2-low asthma, and ACO, are clinical-molecular composite phenotypes rather than pure molecular endotypes, and each retains internal heterogeneity. We present this classification not as a definitive endpoint but as an operational minimal framework: each stratum carries a distinct weighting of the mitochondrion–NETosis axis (**Table 1**) and maps onto a different actionable therapeutic node, from type-2-cytokine blockade in type-2-high disease to NET-directed and mitochondria-directed strategies in type-2-low and COPD, and combination approaches in ACO. The precision-therapeutic goal is then realised by superimposing molecular biomarkers on this phenotypic entry layer, so that patients within any stratum are further enriched by NET-high, mtDNA-high, or interferon-signature-high status before randomisation. Phenotype provides the entry point; biomarker enrichment delivers the precision.

## 7 Therapeutic implications: from NET disruption to mitochondrial precision medicine

### 7.1 Validated scaffold-directed and cytokine-directed therapies

With asthma–COPD overlap exposing the convergent operation of eosinophilic and neutrophilic NETopathy on a shared mitochondrial substrate, the immediate translational question becomes which therapeutic strategies engage this convergent axis in clinical practice, and where the maturity gaps lie. The clinical relevance of the mitochondrion–NETosis axis as target is established by inhaled rhDNase, standard-of-care in cystic fibrosis. In the phase III trial of 968 patients, once- and twice-daily rhDNase reduced age-adjusted exacerbation risk by 28% (p = 0.04) and 37% (p < 0.01), respectively, with FEV_1_ improvements ~5.6%-5.8% (116). The principle of direct extracellular-DNA-scaffold disruption is reinforced by §2.3 and §2.4: DNase I restored glucocorticoid sensitivity in cigarette-smoke COPD models, inhibited airway hyperreactivity in neutrophilic asthma where corticosteroids failed, and protected mice from rhinovirus-induced type-2 immunopathology. The contemporary armamentarium for severe type-2-high asthma rests on monoclonal antibodies neutralising IL-5, IL-5Rα, IL-4Rα, or TSLP. Mepolizumab reduced annualised exacerbation rates by 47% (IV) to 53% (SC) in the MENSA trial of 576 patients (p < 0.001) (117) and enabled a median 50% oral-glucocorticoid dose reduction in the SIRIUS trial of 135 patients, with 32% exacerbation-rate reduction despite taper (118). Benralizumab, an afucosylated anti-IL-5Rα antibody depleting eosinophils through antibody-dependent cellular cytotoxicity, reduced annual exacerbation rates by 36% (Q4W) and 28% (Q8W) in the CALIMA phase 3 trial of 1,306 patients stratified by blood eosinophils (119). The ABRA trial of 158 patients with acute eosinophilic exacerbations of asthma and COPD found a single benralizumab with or without standard-of-care prednisolone reduced treatment failure at 90 days from 74% (prednisolone alone) to 45% (pooled benralizumab; OR 0.26, 95% CI 0.13-0.56, p = 0.0005), with a 28-day visual-analogue-scale symptom advantage of 49 mm (120). Tezepelumab, anti-TSLP, demonstrated efficacy across the entire blood-eosinophil spectrum in the NAVIGATOR phase 3 trial of 1,061 patients: annualised exacerbation rate 0.93 versus 2.10 with placebo (rate ratio 0.44, 95% CI 0.37-0.53, p < 0.001); in the type-2-low subgroup (<300 eosinophils/μL), the rate ratio remained 0.59 (95% CI 0.46-0.75, p < 0.001) (121). Dupilumab, anti-IL-4Rα, achieved 70.1% oral-glucocorticoid dose reduction versus 41.9% with placebo in LIBERTY ASTHMA VENTURE (210 steroid-dependent patients), with 48% versus 25% discontinuing oral corticosteroid entirely and a 59% severe-exacerbation reduction despite reduced steroid (122). The biologic toolkit leaves a substantial residual, type-2-low neutrophilic asthma, severe COPD without eosinophilic exacerbations, and the corticosteroid-resistant neutrophilic phenotype from Chapters 2.3 and 2.4 for which the mitochondrion–NETosis axis offers an alternative entry.

### 7.2 Mitochondrial-targeted antioxidants and neutrophil-axis targeting

The mitochondria-targeted antioxidant (MTA) concept, lipophilic triphenylphosphonium conjugated to an antioxidant for several-hundred-fold mitochondrial accumulation through inner-membrane potential, was articulated by Murphy (123). MitoQ passes plasma and inner mitochondrial membranes including the blood–brain barrier, providing a tractable approach to mtROS pathology. Fairley summarised oxidative stress as a major COPD hallmark driving inflammation, corticosteroid resistance, DNA damage, lung aging, and senescence, with COPD-lung mitochondria exhibiting impaired structure function and reduced oxidative capacity, and MTAs under development as more focused antioxidant therapy (124). Agrawal and Mabalirajan documented MitoQ substantially improving mitochondrial health and respiratory function in experimental COPD and asthma, with non-coding RNA modulation of biogenesis/dynamics/degradation and healthy-mitochondrial transfer into epithelial cells also efficacious (125). Caldeira placed mitochondrial morphology, dynamics, redox, mitophagy, and ER cross-talk as central nodes across COPD, IPF, and asthma (126). Barnes’s synthesis established that oxidative stress in COPD reduces Nrf2 and FOXO defences, contributes to skeletal-muscle weakness, and amplifies inflammation, fibrosis, emphysema, corticosteroid resistance, lung aging, and autoantibody formation (127). Cen showed that MitoQ reduced LPS-induced ROS, mitochondrial dysfunction, and endothelial-cell apoptosis in human pulmonary microvascular endothelial cells and ameliorated acute lung injury in mice (128). PGC-1α, the master mitochondrial-biogenesis regulator linked to cardiac dysfunction, is a candidate therapeutic node (129). In the asthma airway, MitoQ reduced eotaxin, eosinophilia, inflammation, remodelling, and hyperresponsiveness in obese house-dust-mite mice. Direct neutrophil targeting through CXCR2 antagonism blocks IL-8/CXCL1-dependent recruitment without depleting the systemic neutrophil pool. In the Lazaar Phase 2b trial, 614 patients with mild-to-moderate COPD received danirixin at 5 to 50 mg twice daily or placebo for 26 weeks, and there was no improvement in respiratory-symptom, CAT, or St George’s Respiratory Questionnaire scores at any dose. More troubling than the lack of benefit, exacerbations were more frequent on danirixin, with 56 to 75 events across the dose arms against 44 on placebo, the time to first exacerbation shortened from 110 days on placebo to between 47 and 79 days, pneumonia rose to 5% at the 50-mg dose against under 1% on placebo, and all five on-treatment deaths occurred in danirixin arms (130). The Keir mechanistic reanalysis of the same trial identified the failure as stratified: COPD neutrophil activation and NETosis operate through both CXCL8/IL-8-dependent and -independent pathways, and CXCR2 antagonism is biologically effective only in IL-8-dominant patients reframing the disappointing aggregate result as patient mis-selection rather than mechanism refutation (131). Complementary lines target the elastase pathway through GW311616A, preventing cigarette-smoke-induced NETs and attenuating airway pathology, and PAD4 inhibition with Cl-amidine and related compounds blocking chromatin citrullination upstream of NETs. The structural consequences, airway-wall remodelling, smooth-muscle hypertrophy, collagen deposition, and subepithelial fibrosis, are longer-term endpoints towards which the axis drives. Liu et al. mapped remodelling features across asthma, COPD, and IPF, with both common and compartment-specific extracellular-matrix dysregulation, arguing that airway versus parenchymal strategies will differ in molecular target and delivery (132). **Table 2** consolidates these five therapeutic axes within the mitochondrion–NETosis framework and highlights a deliberate maturity asymmetry: NET dissolution and upstream-cytokine biologics are clinically validated but endotype-restricted, whereas mitochondrial-targeted antioxidants and metabolic–epigenetic strategies address the convergent root yet remain without completed RCTs. The asymmetry defines the central translational gap. Closing this gap depends, first, on resolving the mechanistic uncertainties that complicate decisions about which arm of the cycle to drug, and only then on the cross-disciplinary translational architecture by which more mature fields have already navigated analogous decisions (**Figure 3**, **Table 2**).

The danirixin experience, and the preclinical status of the mitochondrial and metabolic-epigenetic regulators, raises the legitimate question of whether any intervention that does not explicitly target a pathogenic neutrophil subpopulation can succeed. We suggest that mechanism-directed agents carry an intrinsic, if imperfect, form of subset selectivity: because the mtROS source, the mPTP, oxidised-mtDNA release, PAD4 citrullination, and gasdermin pores are engaged predominantly in neutrophils that have undergone pathological mitochondrial reprogramming, inhibiting these nodes preferentially disables the NET-competent disease subset whilst largely sparing the quiescent circulating and marginated reservoir required for antimicrobial defence. This stands in contrast to indiscriminate neutrophil depletion or broad chemotaxis blockade, which remove protective and pathogenic cells alike. Subset-directed strategies represent the next refinement: the PROK2-positive COPD subset and the low-density granulocyte population expanded in severe asthma offer surface or secreted markers through which pathogenic cells could in principle be selectively modulated or depleted (49, 50). The therapeutic window is therefore defined not by maximal neutrophil suppression but by selective silencing of the reprogrammed subset, preserving the basal neutrophil functions on which airway host defence depends.

## 8 Discussion

This review has argued that the neutrophil mitochondrion is a four-faceted upstream switch: an mtROS source linked to HIF-1α and glycolytic-flux control, an mPTP-gated arbitrator of calcium and membrane integrity, a factory for mtDNA-DAMP release, and a substrate for PINK1/Parkin-mediated clearance and PGC-1α-driven biogenesis whose disruption licenses sustained NETosis across the neutrophil-dominated phenotype of COPD, the endotype-stratified NETs/EETs of severe asthma, and the convergent neutrophilic final common pathway of ACO that carries the highest mortality burden of the three (115). The disease-specific upstream drivers (smoke-driven ferroptosis resistance through Nrf2–SLC7A11–GPX4 (84); IL-33/IL-17/TSLP-driven cellular licensing (96); T2D- and hyperglycaemia-primed dysfunction) reweight rather than replace a shared mtROS–mtDNA–NET axis, recasting the three legacy diagnoses as molecular-phenotype subgroups defined by treatable traits. The asymmetry shown in **Table 2** makes explicit proven distal interventions coexisting with mechanistically aligned but clinically immature proximal interventions, which is the predictable signature of a field that has matured cytokine-target neutralisation faster than mechanism-anchored intervention and motivates the mechanism-then-translation questions that follow.

The mechanistic paradigm that has consolidated treats the neutrophil mitochondrion as a quantitative gate rather than a binary switch: mtROS through the glycerol-3-phosphate shuttle stabilises HIF-1α and sustains NET-licensing transcription, oxidised mtDNA released after mitochondrial damage activates cGAS–STING and TLR9 in a self-amplifying autocrine loop, gasdermin D and gasdermin E pores execute the permeabilisation step shared with pyroptosis, ferroptosis-resistant survival through Nrf2–SLC7A11–GPX4 sustains NET output despite dexamethasone in steroid-resistant COPD neutrophils, and m6A methylation of HIF-1α and GPX4 reprograms mitochondrial metabolism downstream of NETs to perpetuate the cycle, a modality spectrum converging on shared mitochondrial gates that distinguishes infection-driven release from the steroid-resistant, mtDNA-rich phenotypes dominating chronic airway disease (133, 134). The honest concession is that this paradigm rests on associative human evidence reinforced by mouse genetic and patient-cell rescue. Whether oxidised mtDNA threaded through NETs causally drives the type I interferon programme settled in lupus where mtDNA-bearing NETs reproduce the signature on transfer but only correlative in COPD and severe asthma remains open, as does whether mitophagy failure is the founding lesion along the PINK1/Parkin axis or itself an mtROS-driven downstream collapse. The methodological infrastructure to settle both is now in hand: single-cell neutrophil endotyping operationalised in lung injury cohorts is mature enough to port into outpatient sputum (135, 136), BAL, and bronchial-biopsy sampling; longitudinal multi-omics measured before and after exacerbation would resolve mitochondrial NETopathy into temporally and spatially stratified states (137, 138); and cell-free mitochondrial DNA, already prognostic for 28-day mortality in septic shock (139), harmonised against airway cohorts providing the exacerbation-phase damage biomarker whose dynamics can assign causality to oxidised mtDNA.

A related methodological caveat concerns the cellular resolution of the evidence base. The correlations linking mitochondrial dysfunction to corticosteroid resistance in COPD, severe asthma, and ACO derive predominantly from bulk quantification of mixed cell populations in sputum and bronchoalveolar lavage fluid, in which neutrophil-derived signals are averaged against epithelial, macrophage, and lymphoid contributions and are therefore vulnerable to cellular-composition confounding. Single-cell resolution of airway neutrophils is only now becoming available: single-cell RNA-sequencing of COPD sputum and lung tissue has begun to resolve neutrophil-enriched endotypes with exaggerated inflammatory signalling (140), machine-learning integration of single-cell and bulk transcriptomes has derived NET-related gene models that stratify COPD subtypes (141), and single-cell profiling of asthma neutrophils is identifying subpopulations whose proportions shift with disease severity. We therefore regard the bulk biomarkers discussed throughout this review, sputum extracellular DNA, NET-protein complexes, and cell-free mitochondrial DNA, as clinically valuable precisely because they are accessible, quantitative, and deployable at the bedside for patient stratification, whilst emphasising that their mechanistic attribution to specific neutrophil subpopulations requires confirmation by single-cell and spatial approaches. A pragmatic two-tier strategy follows: bulk panels for screening and enrichment in trials, with single-cell endotyping reserved for mechanistic confirmation and biomarker validation.

Several limitations temper this framework and define the work still needed. Because NETs evolved as antimicrobial structures, therapy that dissolves or prevents them risks weakening defence against the pathogens that drive exacerbations such as Staphylococcus aureus, rhinovirus, Aspergillus, and Pseudomonas, so the therapeutic window is set by balance rather than by maximal suppression, and the safest route is to remove the pathological NET burden selectively with scaffold-directed or interferon-loop-directed agents rather than to shut NET formation down globally (142–144). The proposed contribution of the axis to corticosteroid resistance is also best read as complementary to the established multifactorial mechanisms, amongst them reduced HDAC2 activity, an elevated dominant-negative glucocorticoid-receptor β isoform, and NLRP3, IL-17/Th17, p38-MAPK, and microRNA-21/PI3K signalling, with mtROS positioned as a plausible upstream driver of HDAC2 loss rather than as a replacement for these pathways (145–147). Finally, several facets of mitochondrial reprogramming remain only lightly explored here, including the fission and fusion machinery of DRP1, MFN1/2, and OPA1 whose manipulation both licenses and suppresses NET formation (35, 148), the colony-stimulating-factor priming that sets the mitochondrial state permitting vital mtDNA-rich release (149), and the sex differences by which male neutrophils carry stronger mitochondrial and interferon programmes whereas 17β-oestradiol suppresses excess NETs (150, 151).

Translation into therapy will build on a clinical platform mature on the distal end and immature on the proximal end. NET dissolution with nebulised rhDNase has 30 years of cystic fibrosis safety and efficacy data, and upstream T2-cytokine blockade has accumulated pivotal evidence in eosinophilic asthma: mepolizumab in MENSA, benralizumab in CALIMA and ABRA acute augmentation, dupilumab in LIBERTY ASTHMA VENTURE, and tezepelumab in NAVIGATOR. The CXCR2 antagonism story crystallises the gap: the disappointing aggregate result for danirixin in unselected COPD was reframed by biomarker-stratified re-analysis showing benefit confined to IL-8/CXCL8-dependent NET-high patients, converting a failed unselected trial into a positive enrichment signal and exposing the proximal arm, mtROS source, NET assembly, NET-DNA scaffold, and NET-receptor signalling, as still without its MENSA-equivalent pivotal data. The forward agenda is concrete. Harmonisation of a coordinated biomarker panel capturing NET burden, neutrophilic inflammation, and mitochondrial dysfunction in one sampling event, sputum eDNA and NET-protein complexes, blood CCL4L2 in corticosteroid non-response, the CLCA1-NAMPT eosinophilic-versus-neutrophilic model at AUC 0.903 (109), the TNFAIP3-CXCR4-PADI4 severe-asthma panel, and emerging mtDNA assays is the gating trial-readiness step. Phenotype-enriched trials randomising NET-high, mtDNA-high, or interferon-signature-high patients to NET-directed (DNase I, PAD4, and NE), mitochondria-directed, or combination arms with adaptive biomarker-guided randomisation are the architecture required, with explicit ACO basket-arm inclusion since that highest-mortality subgroup remains systematically under-represented. Such a trial would enrich for NET-high, mtDNA-high, interferon-signature-high patients and measure paired sputum and bronchoalveolar-lavage NET burden, cell-free mtDNA, oxidised-mtDNA fraction, and an interferon-stimulated-gene score both before and during an exacerbation. A falling interferon signature that tracks a falling oxidised-mtDNA burden under mitochondria-directed therapy but not under scaffold dissolution alone would convert the present correlative claim into a causal one. Inhaled airway-targeted delivery of the mitochondrial pharmacophore is the complementary engineering priority that would convert the proximal end of the axis from a preclinical concept into a clinical asset.

A further design consideration for the proposed trials is that the agents under discussion are not taken up uniformly across airway cell types. Mitochondria-targeted antioxidants such as MitoQ accumulate several-hundred-fold within mitochondria in proportion to membrane potential, so epithelial cells, airway smooth muscle, alveolar macrophages, and neutrophils, which differ markedly in mitochondrial content and membrane potential, will achieve very different intracellular concentrations of the same administered dose. This differential uptake cuts both ways: it complicates dose selection when the intended target is the neutrophil, but it also offers an engineering opportunity. Neutrophil-directed delivery strategies are advancing rapidly, including neutrophil-membrane-coated and neutrophil-hitchhiking nanoparticles that exploit the cells’ own tropism for inflamed tissue (152, 153), and respiratory delivery of neutrophil-specific liposomes that suppress NET formation directly within the lung (154). Inhaled administration of the mitochondrial pharmacophore would additionally concentrate drug at the airway surface where the pathogenic neutrophils reside. Future phenotype-enriched trials should therefore incorporate cell-type-resolved pharmacodynamic readouts, for example mtROS or oxidised-mtDNA measured in flow-sorted airway neutrophils, rather than relying solely on whole-sputum or whole-lavage measurements, so that target engagement in the intended cell can be demonstrated directly.

## 9 Conclusion

The neutrophil mitochondrion has moved from unexamined background organelle to the upstream switch that licenses sustained NETosis across the corticosteroid-resistant phenotypes of COPD, severe asthma, and asthma–COPD overlap. Disease-specific drivers reweight a shared mtROS–mtDNA–NET axis whose individual nodes, mtROS source, NET assembly, mtDNA-DAMP scaffold, and NET-receptor signalling define a tractable framework of treatable traits. Translating that framework into care depends on a coordinated biomarker panel deployable at the bedside, phenotype-enriched trials randomising molecularly selected patients rather than diagnostic categories, and explicit inclusion of the asthma–COPD overlap subgroup. The proximal arm of the axis still awaits its pivotal-data moment; the framework, the targets, and the stratification logic to deliver it are now defined and testable.

## References

[1] Adeloye D Song P Zhu Y Campbell H Sheikh A Rudan I . Global, regional, and national prevalence of, and risk factors for, chronic obstructive pulmonary disease (COPD) in 2019: A systematic review and modelling analysis. Lancet Respir Med. (2022) 10:447–58. doi: 10.1016/S2213-2600(21)00511-7 35279265 PMC9050565

[2] Venkatesan P . GOLD COPD report: 2024 update. Lancet Respir Med. (2024) 12:15–6. doi: 10.1016/S2213-2600(23)00461-7 38061380

[3] Israel E Reddel HK . Severe and difficult-to-treat asthma in adults. N Engl J Med. (2017) 377:965–76. doi: 10.1056/NEJMra1608969 28877019

[4] Hosseini M Almasi-Hashiani A Sepidarkish M Maroufizadeh S . Global prevalence of asthma-COPD overlap (ACO) in the general population: A systematic review and meta-analysis. Respir Res. (2019) 20:229. doi: 10.1186/s12931-019-1198-4 31647021 PMC6813073

[5] Zhu M Chen A . Epidemiological characteristics of asthma-COPD overlap, its association with all-cause mortality, and the mediating role of depressive symptoms: Evidence from NHANES 2005-2018. BMC Public Health. (2024) 24:1423. doi: 10.1186/s12889-024-18911-1 38807148 PMC11134654

[6] Mattila T Vasankari T Kauppi P Mazur W Härkänen T Heliövaara M . Mortality of asthma, COPD, and asthma-COPD overlap during an 18-year follow up. Respir Med. (2023) 207:107112. doi: 10.1016/j.rmed.2022.107112 36596385

[7] Brinkmann V Reichard U Goosmann C Fauler B Uhlemann Y Weiss DS . Neutrophil extracellular traps kill bacteria. Science. (2004) 303:1532–5. doi: 10.1126/science.1092385 15001782

[8] Wang H Kim SJ Lei Y Wang S Wang H Huang H . Neutrophil extracellular traps in homeostasis and disease. Signal Transduct Target Ther. (2024) 9:235. doi: 10.1038/s41392-024-01933-x 39300084 PMC11415080

[9] Papayannopoulos V . Neutrophil extracellular traps in immunity and disease. Nat Rev Immunol. (2018) 18:134–47. doi: 10.1038/nri.2017.105 28990587

[10] Hammad H Lambrecht BN . The basic immunology of asthma. Cell. (2021) 184:2521–2. doi: 10.1016/j.cell.2021.04.019 33930297

[11] Torfs K Vermeersch G Gouwy M Devos T Proost P Struyf S . Neutrophils as critical orchestrators of chronic inflammation. Cell Mol Immunol. (2026) 23:123–49. doi: 10.1038/s41423-025-01380-w 41530536 PMC12858905

[12] Cao Z Zhao M Sun H Hu L Chen Y Fan Z . Roles of mitochondria in neutrophils. Front Immunol. (2022) 13:934444. doi: 10.3389/fimmu.2022.934444 36081497 PMC9447286

[13] Willson JA Arienti S Sadiku P Reyes L Coelho P Morrison T . Neutrophil HIF-1α stabilization is augmented by mitochondrial ROS produced via the glycerol 3-phosphate shuttle. Blood. (2022) 139:281–6. doi: 10.1182/blood.2021011010 34411229 PMC8832465

[14] Amulic B Cazalet C Hayes GL Metzler KD Zychlinsky A . Neutrophil function: From mechanisms to disease. Annu Rev Immunol. (2012) 30:459–89. doi: 10.1146/annurev-immunol-020711-074942 22224774

[15] Borregaard N . Neutrophils, from marrow to microbes. Immunity. (2010) 33:657–70. doi: 10.1016/j.immuni.2010.11.011 21094463

[16] Maldarelli ME Noto MJ . The emerging role for neutrophil mitochondrial metabolism in lung inflammation. Immunometabolism (Cobham). (2024) 6:e00036. doi: 10.1097/IN9.0000000000000036 38283697 PMC10810349

[17] West AP Khoury-Hanold W Staron M Tal MC Pineda CM Lang SM . Mitochondrial DNA stress primes the antiviral innate immune response. Nature. (2015) 520:553–7. doi: 10.1038/nature14156 25642965 PMC4409480

[18] Narendra DP Youle RJ . The role of PINK1-parkin in mitochondrial quality control. Nat Cell Biol. (2024) 26:1639–51. doi: 10.1038/s41556-024-01513-9 39358449

[19] Fricker M Lokwani R . COPD: The role of neutrophils in inflammation, pathophysiology, and as drug targets. Clin Sci (Lond). (2025) 139:1199–214. doi: 10.1042/CS20255452 41124071 PMC12687445

[20] Morán G Uberti B Quiroga J . Role of cellular metabolism in the formation of neutrophil extracellular traps in airway diseases. Front Immunol. (2022) 13:850416. doi: 10.3389/fimmu.2022.850416 35493475 PMC9039247

[21] Lam HC Cloonan SM Bhashyam AR Haspel JA Singh A Sathirapongsasuti JF . Histone deacetylase 6-mediated selective autophagy regulates COPD-associated cilia dysfunction. J Clin Invest. (2013) 123:5212–30. doi: 10.1172/JCI69636 24200693 PMC3859407

[22] Azzouz D Palaniyar N . How do ROS induce NETosis? Oxidative DNA damage, DNA repair, and chromatin decondensation. Biomolecules. (2024) 14:1307. doi: 10.3390/biom14101307 39456240 PMC11505619

[23] Xia L Yan X Zhang H . Mitochondrial DNA-activated cGAS-STING pathway in cancer: Mechanisms and therapeutic implications. Biochim Biophys Acta (BBA) - Rev Cancer. (2025) 1880:189249. doi: 10.1016/j.bbcan.2024.189249 39701325

[24] Vorobjeva NV Chernyak BV . NETosis: Molecular mechanisms, role in physiology and pathology. Biochem (Mosc). (2020) 85:1178–90. doi: 10.1134/S0006297920100065 33202203 PMC7590568

[25] Blaustein MP Lederer WJ . Sodium/calcium exchange: Its physiological implications. Physiol Rev. (1999) 79:763–854. doi: 10.1152/physrev.1999.79.3.763 10390518

[26] Leblanc P-O Bourgoin SG Poubelle PE Tessier PA Pelletier M . Metabolic regulation of neutrophil functions in homeostasis and diseases. J Leukoc Biol. (2024) 116:456–68. doi: 10.1093/jleuko/qiae025 38452242

[27] Mizumura K Cloonan SM Nakahira K Bhashyam AR Cervo M Kitada T . Mitophagy-dependent necroptosis contributes to the pathogenesis of COPD. J Clin Invest. (2014) 124:3987–4003. doi: 10.1172/JCI74985 25083992 PMC4151233

[28] Tsubouchi K Araya J Kuwano K . PINK1-PARK2-mediated mitophagy in COPD and IPF pathogeneses. Inflammation Regener. (2018) 38:18. doi: 10.1186/s41232-018-0077-6 30386443 PMC6199723

[29] Araya J Tsubouchi K Sato N Ito S Minagawa S Hara H . PRKN-regulated mitophagy and cellular senescence during COPD pathogenesis. Autophagy. (2019) 15:510–26. doi: 10.1080/15548627.2018.1532259 30290714 PMC6351145

[30] Lika J Votava JA Datta R Mellado Fritz CA Kralovec AM Smith FM . Mitochondrial metabolism is rapidly re-activated in mature neutrophils to support stimulation-induced response. Front Immunol. (2025) 16:1572927. doi: 10.3389/fimmu.2025.1572927 40356902 PMC12066771

[31] Ito S Araya J Kurita Y Kobayashi K Takasaka N Yoshida M . PARK2-mediated mitophagy is involved in regulation of HBEC senescence in COPD pathogenesis. Autophagy. (2015) 11:547–59. doi: 10.1080/15548627.2015.1017190 25714760 PMC4502689

[32] Abu Shelbayeh O Arroum T Morris S Busch KB . PGC-1α is a master regulator of mitochondrial lifecycle and ROS stress response. Antioxidants (Basel). (2023) 12:1075. doi: 10.3390/antiox12051075 37237941 PMC10215733

[33] Qian L Zhu Y Deng C Liang Z Chen J Chen Y . Peroxisome proliferator-activated receptor gamma coactivator-1 (PGC-1) family in physiological and pathophysiological process and diseases. Signal Transduct Target Ther. (2024) 9:50. doi: 10.1038/s41392-024-01756-w 38424050 PMC10904817

[34] Brinkmann V Zychlinsky A . Neutrophil extracellular traps: Is immunity the second function of chromatin? J Cell Biol. (2012) 198:773–83. doi: 10.1083/jcb.201203170 22945932 PMC3432757

[35] Boeltz S Amini P Anders H-J Andrade F Bilyy R Chatfield S . To NET or not to NET:Current opinions and state of the science regarding the formation of neutrophil extracellular traps. Cell Death Differ. (2019) 26:395–408. doi: 10.1038/s41418-018-0261-x 30622307 PMC6370810

[36] Lood C Blanco LP Purmalek MM Carmona-Rivera C De Ravin SS Smith CK . Neutrophil extracellular traps enriched in oxidized mitochondrial DNA are interferogenic and contribute to lupus-like disease. Nat Med. (2016) 22:146–53. doi: 10.1038/nm.4027 26779811 PMC4742415

[37] Yousefi S Mihalache C Kozlowski E Schmid I Simon HU . Viable neutrophils release mitochondrial DNA to form neutrophil extracellular traps. Cell Death Differ. (2009) 16:1438–44. doi: 10.1038/cdd.2009.96 19609275

[38] Fuchs TA Abed U Goosmann C Hurwitz R Schulze I Wahn V . Novel cell death program leads to neutrophil extracellular traps. J Cell Biol. (2007) 176:231–41. doi: 10.1083/jcb.200606027 17210947 PMC2063942

[39] Papayannopoulos V Metzler KD Hakkim A Zychlinsky A . Neutrophil elastase and myeloperoxidase regulate the formation of neutrophil extracellular traps. J Cell Biol. (2010) 191:677–91. doi: 10.1083/jcb.201006052 20974816 PMC3003309

[40] Hakkim A Fuchs TA Martinez NE Hess S Prinz H Zychlinsky A . Activation of the raf-MEK-ERK pathway is required for neutrophil extracellular trap formation. Nat Chem Biol. (2011) 7:75–7. doi: 10.1038/nchembio.496 21170021

[41] Wang Y Li M Stadler S Correll S Li P Wang D . Histone hypercitrullination mediates chromatin decondensation and neutrophil extracellular trap formation. J Cell Biol. (2009) 184:205–13. doi: 10.1083/jcb.200806072 19153223 PMC2654299

[42] Lewis HD Liddle J Coote JE Atkinson SJ Barker MD Bax BD . Inhibition of PAD4 activity is sufficient to disrupt mouse and human NET formation. Nat Chem Biol. (2015) 11:189–91. doi: 10.1038/nchembio.1735 25622091 PMC4397581

[43] Thiam HR Wong SL Qiu R Kittisopikul M Vahabikashi A Goldman AE . NETosis proceeds by cytoskeleton and endomembrane disassembly and PAD4-mediated chromatin decondensation and nuclear envelope rupture. Proc Natl Acad Sci USA. (2020) 117:7326–37. doi: 10.1073/pnas.1909546117 32170015 PMC7132277

[44] Granton E Kim JH Podstawka J Yipp BG . The lung microvasculature is a functional immune niche. Trends Immunol. (2018) 39:890–9. doi: 10.1016/j.it.2018.09.002 30253910

[45] Yipp BG Kim JH Lima R Zbytnuik LD Petri B Swanlund N . The lung is a host defense niche for immediate neutrophil-mediated vascular protection. Sci Immunol. (2017) 2:eaam8929. doi: 10.1126/sciimmunol.aam8929 28626833 PMC5472445

[46] Summers C Rankin SM Condliffe AM Singh N Peters AM Chilvers ER . Neutrophil kinetics in health and disease. Trends Immunol. (2010) 31:318–24. doi: 10.1016/j.it.2010.05.006 20620114 PMC2930213

[47] Grieshaber-Bouyer R Radtke FA Cunin P Stifano G Levescot A Vijaykumar B . The neutrotime transcriptional signature defines a single continuum of neutrophils across biological compartments. Nat Commun. (2021) 12:2856. doi: 10.1038/s41467-021-22973-9 34001893 PMC8129206

[48] Zhang D Chen G Manwani D Mortha A Xu C Faith JJ . Neutrophil ageing is regulated by the microbiome. Nature. (2015) 525:528–36. doi: 10.1038/nature15367 26374999 PMC4712631

[49] Li R Cheng C Zhao X Xu H Fei Y Zhao S . PROK2 defines a pathogenic neutrophil subset and serves as a clinical indicator of neutrophilic inflammation in COPD. Respir Res. (2026) 27:239. doi: 10.1186/s12931-026-03679-2 41998641 PMC13267660

[50] Fu J Tobin MC Thomas LL . Neutrophil-like low-density granulocytes are elevated in patients with moderate to severe persistent asthma. Ann Allergy Asthma Immunol. (2014) 113:635–40.e2. doi: 10.1016/j.anai.2014.08.024 25256681

[51] Wang K Wang M Liao X Gao S Hua J Wu X . Locally organised and activated Fth1hi neutrophils aggravate inflammation of acute lung injury in an IL-10-dependent manner. Nat Commun. (2022) 13:7703. doi: 10.1038/s41467-022-35492-y 36513690 PMC9745290

[52] Sollberger G Choidas A Burn GL Habenberger P Di Lucrezia R Kordes S . Gasdermin D plays a vital role in the generation of neutrophil extracellular traps. Sci Immunol. (2018) 3:eaar6689. doi: 10.1126/sciimmunol.aar6689 30143555

[53] Zhu YP Speir M Tan Z Lee JC Nowell CJ Chen AA . NET formation is a default epigenetic program controlled by PAD4 in apoptotic neutrophils. Sci Adv. (2023) 9:eadj1397. doi: 10.1126/sciadv.adj1397 38117877 PMC10732518

[54] Yipp BG Petri B Salina D Jenne CN Scott BNV Zbytnuik LD . Infection-induced NETosis is a dynamic process involving neutrophil multitasking *in vivo*. Nat Med. (2012) 18:1386–93. doi: 10.1038/nm.2847 22922410 PMC4529131

[55] Douda DN Khan MA Grasemann H Palaniyar N . SK3 channel and mitochondrial ROS mediate NADPH oxidase-independent NETosis induced by calcium influx. Proc Natl Acad Sci USA. (2015) 112:2817–22. doi: 10.1073/pnas.1414055112 25730848 PMC4352781

[56] Wong SL Demers M Martinod K Gallant M Wang Y Goldfine AB . Diabetes primes neutrophils to undergo NETosis, which impairs wound healing. Nat Med. (2015) 21:815–9. doi: 10.1038/nm.3887 26076037 PMC4631120

[57] Liu L Mao Y Xu B Zhang X Fang C Ma Y . Induction of neutrophil extracellular traps during tissue injury: Involvement of STING and toll-like receptor 9 pathways. Cell Prolif. (2019) 52:e12579. doi: 10.1111/cpr.12579 30851061 PMC6536408

[58] Mallavia B Liu F Lefrançais E Cleary SJ Kwaan N Tian JJ . Mitochondrial DNA stimulates TLR9-dependent neutrophil extracellular trap formation in primary graft dysfunction. Am J Respir Cell Mol Biol. (2020) 62:364–72. doi: 10.1165/rcmb.2019-0140OC 31647878 PMC7055700

[59] Aubé F-A Bidias A Pépin G . Who and how, DNA sensors in NETs-driven inflammation. Front Immunol. (2023) 14:1190177. doi: 10.3389/fimmu.2023.1190177 37187738 PMC10179500

[60] Li Y Hook JS Ding Q Xiao X Chung SS Mettlen M . Neutrophil metabolomics in severe COVID-19 reveal GAPDH as a suppressor of neutrophil extracellular trap formation. Nat Commun. (2023) 14:2610. doi: 10.1038/s41467-023-37567-w 37147288 PMC10162006

[61] Zhang H Wu D Wang Y Guo K Spencer CB Ortoga L . METTL3-mediated N6-methyladenosine exacerbates ferroptosis via m6A-IGF2BP2-dependent mitochondrial metabolic reprogramming in sepsis-induced acute lung injury. Clin Transl Med. (2023) 13:e1389. doi: 10.1002/ctm2.1389 37715457 PMC10504453

[62] Zhang H Liu J Zhou Y Qu M Wang Y Guo K . Neutrophil extracellular traps mediate m6A modification and regulates sepsis-associated acute lung injury by activating ferroptosis in alveolar epithelial cells. Int J Biol Sci. (2022) 18:3337–57. doi: 10.7150/ijbs.69141 35637949 PMC9134924

[63] Qu M Chen Z Qiu Z Nan K Wang Y Shi Y . Neutrophil extracellular traps-triggered impaired autophagic flux via METTL3 underlies sepsis-associated acute lung injury. Cell Death Discov. (2022) 8:375. doi: 10.1038/s41420-022-01166-3 36030287 PMC9420153

[64] Sule G Abuaita BH Steffes PA Fernandes AT Estes SK Dobry C . Endoplasmic reticulum stress sensor IRE1α propels neutrophil hyperactivity in lupus. J Clin Invest. (2021) 131:e137866, 137866. doi: 10.1172/JCI137866 33561013 PMC8011900

[65] Pérez-Figueroa E Álvarez-Carrasco P Ortega E Maldonado-Bernal C . Neutrophils: Many ways to die. Front Immunol. (2021) 12:631821. doi: 10.3389/fimmu.2021.631821 33746968 PMC7969520

[66] Thiam HR Wong SL Wagner DD Waterman CM . Cellular mechanisms of NETosis. Annu Rev Cell Dev Biol. (2020) 36:191–218. doi: 10.1146/annurev-cellbio-020520-111016 32663035 PMC8499668

[67] Castanheira FVS Kubes P . Neutrophils and NETs in modulating acute and chronic inflammation. Blood. (2019) 133:2178–85. doi: 10.1182/blood-2018-11-844530 30898862

[68] Katsoulis O Toussaint M Jackson MM Mallia P Footitt J Mincham KT . Neutrophil extracellular traps promote immunopathogenesis of virus-induced COPD exacerbations. Nat Commun. (2024) 15:5766. doi: 10.1038/s41467-024-50197-0 38982052 PMC11233599

[69] Zeng Q Xue L Li W Liang C Zhou W Xiong W . Inhibition of 5-hydroxyindoleacetic acid to reduce neutrophil extracellular trap production improves lung condition in chronic obstructive pulmonary disease mice. Ann Med. (2025) 57:2474734. doi: 10.1080/07853890.2025.2474734 40066951 PMC11899248

[70] Fousert E Toes R Desai J . Neutrophil extracellular traps (NETs) take the central stage in driving autoimmune responses. Cells. (2020) 9:915. doi: 10.3390/cells9040915 32276504 PMC7226846

[71] Xian H Watari K Sanchez-Lopez E Offenberger J Onyuru J Sampath H . Oxidized DNA fragments exit mitochondria via mPTP- and VDAC-dependent channels to activate NLRP3 inflammasome and interferon signaling. Immunity. (2022) 55:1370–85.e8. doi: 10.1016/j.immuni.2022.06.007 35835107 PMC9378606

[72] Kim J Kim H-S Chung JH . Molecular mechanisms of mitochondrial DNA release and activation of the cGAS-STING pathway. Exp Mol Med. (2023) 55:510–9. doi: 10.1038/s12276-023-00965-7 36964253 PMC10037406

[73] Giordano L Ware SA Lagranha CJ Kaufman BA . Mitochondrial DNA signals driving immune responses: Why, how, where? Cell Commun Signal. (2025) 23:192. doi: 10.1186/s12964-025-02042-0 40264103 PMC12012978

[74] Hogg JC Chu F Utokaparch S Woods R Elliott WM Buzatu L . The nature of small-airway obstruction in chronic obstructive pulmonary disease. N Engl J Med. (2004) 350:2645–53. doi: 10.1056/NEJMoa032158 15215480

[75] Hogg JC Paré PD Hackett T-L . The contribution of small airway obstruction to the pathogenesis of chronic obstructive pulmonary disease. Physiol Rev. (2017) 97:529–52. doi: 10.1152/physrev.00025.2015 28151425 PMC6151481

[76] Lareau SC Fahy B Meek P Wang A . Chronic obstructive pulmonary disease (COPD). Am J Respir Crit Care Med. (2019) 199:P1–2. doi: 10.1164/rccm.1991P1 30592446

[77] Brightling C Greening N . Airway inflammation in COPD: Progress to precision medicine. Eur Respir J. (2019) 54:1900651. doi: 10.1183/13993003.00651-2019 31073084

[78] Grabcanovic-Musija F Obermayer A Stoiber W Krautgartner W-D Steinbacher P Winterberg N . Neutrophil extracellular trap (NET) formation characterises stable and exacerbated COPD and correlates with airflow limitation. Respir Res. (2015) 16:59. doi: 10.1186/s12931-015-0221-7 25994149 PMC4455316

[79] Dicker AJ Crichton ML Pumphrey EG Cassidy AJ Suarez-Cuartin G Sibila O . Neutrophil extracellular traps are associated with disease severity and microbiota diversity in patients with chronic obstructive pulmonary disease. J Allergy Clin Immunol. (2018) 141:117–27. doi: 10.1016/j.jaci.2017.04.022 28506850 PMC5751731

[80] Uddin M Watz H Malmgren A Pedersen F . NETopathic inflammation in chronic obstructive pulmonary disease and severe asthma. Front Immunol. (2019) 10:47. doi: 10.3389/fimmu.2019.00047 30804927 PMC6370641

[81] Trivedi A Khan MA Bade G Talwar A . Orchestration of neutrophil extracellular traps (nets), a unique innate immune function during chronic obstructive pulmonary disease (COPD) development. Biomedicines. (2021) 9:53. doi: 10.3390/biomedicines9010053 33435568 PMC7826777

[82] Wiegman CH Michaeloudes C Haji G Narang P Clarke CJ Russell KE . Oxidative stress-induced mitochondrial dysfunction drives inflammation and airway smooth muscle remodeling in patients with chronic obstructive pulmonary disease. J Allergy Clin Immunol. (2015) 136:769–80. doi: 10.1016/j.jaci.2015.01.046 25828268 PMC4559140

[83] Chen J Wang T Li X Gao L Wang K Cheng M . DNA of neutrophil extracellular traps promote NF-κB-dependent autoimmunity via cGAS/TLR9 in chronic obstructive pulmonary disease. Signal Transduct Target Ther. (2024) 9:163. doi: 10.1038/s41392-024-01881-6 38880789 PMC11180664

[84] Wang L Zhu L Tang Y Wen Z Peng L Ci X . Long-term cigarette smoke exposure promotes neutrophil ferroptosis resistance, inducing neutrophil extracellular trap formation and driving glucocorticoid resistance in chronic obstructive pulmonary disease. Res (Wash D C). (2025) 8:751. doi: 10.34133/research.0751 40666830 PMC12260225

[85] Wang K Liao Y Li X Wang R Zeng Z Cheng M . Inhibition of neutrophil elastase prevents cigarette smoke exposure-induced formation of neutrophil extracellular traps and improves lung function in a mouse model of chronic obstructive pulmonary disease. Int Immunopharmacol. (2023) 114:109537. doi: 10.1016/j.intimp.2022.109537 36495695

[86] Keir HR Chalmers JD . Neutrophil extracellular traps in chronic lung disease: Implications for pathogenesis and therapy. Eur Respir Rev. (2022) 31:210241. doi: 10.1183/16000617.0241-2021 35197267 PMC9488971

[87] Wenzel SE . Asthma phenotypes: The evolution from clinical to molecular approaches. Nat Med. (2012) 18:716–25. doi: 10.1038/nm.2678 22561835

[88] Nabe T . Steroid-resistant asthma and neutrophils. Biol Pharm Bull. (2020) 43:31–5. doi: 10.1248/bpb.b19-00095 31902928

[89] Guo H Callaway JB Ting J-Y . Inflammasomes: Mechanism of action, role in disease, and therapeutics. Nat Med. (2015) 21:677–87. doi: 10.1038/nm.3893 26121197 PMC4519035

[90] Xie Y Abel PW Casale TB Tu Y . TH17 cells and corticosteroid insensitivity in severe asthma. J Allergy Clin Immunol. (2022) 149:467–79. doi: 10.1016/j.jaci.2021.12.769 34953791 PMC8821175

[91] Ouyang S Liu C Xiao J Chen X Lui AC Li X . Targeting IL-17A/glucocorticoid synergy to CSF3 expression in neutrophilic airway diseases. JCI Insight. (2020) 5:e132836, 132836. doi: 10.1172/jci.insight.132836 32051346 PMC7098787

[92] Hong L Herjan T Bulek K Xiao J Comhair SAA Erzurum SC . Mechanisms of corticosteroid resistance in type 17 asthma. J Immunol. (2022) 209:1860–9. doi: 10.4049/jimmunol.2200288 36426949 PMC9666330

[93] Lachowicz-Scroggins ME Dunican EM Charbit AR Raymond W Looney MR Peters MC . Extracellular DNA, neutrophil extracellular traps, and inflammasome activation in severe asthma. Am J Respir Crit Care Med. (2019) 199:1076–85. doi: 10.1164/rccm.201810-1869OC 30888839 PMC6515873

[94] Toussaint M Jackson DJ Swieboda D Guedán A Tsourouktsoglou T-D Ching YM . Host DNA released by NETosis promotes rhinovirus-induced type-2 allergic asthma exacerbation. Nat Med. (2017) 23:681–91. doi: 10.1038/nm.4332 28459437 PMC5821220

[95] Curren B Ahmed T Howard DR Ashik Ullah M Sebina I Rashid RB . IL-33-induced neutrophilic inflammation and NETosis underlie rhinovirus-triggered exacerbations of asthma. Mucosal Immunol. (2023) 16:671–84. doi: 10.1016/j.mucimm.2023.07.002 37506849

[96] Tsai C-H Lai A-Y Lin Y-C Chi P-Y Chen Y-C Yang Y-H . Neutrophil extracellular trap production and CCL4L2 expression influence corticosteroid response in asthma. Sci Transl Med. (2023) 15:eadf3843. doi: 10.1126/scitranslmed.adf3843 37285400

[97] Peng B Yan M-Y Chen Y-R Sun F Xiang X-D Liu D . The methyl-CpG binding domain 2 regulates peptidylarginine deiminase 4 expression and promotes neutrophil extracellular trap formation via the janus kinase 2 signaling pathway in experimental severe asthma. Ann Med. (2025) 57:2458207. doi: 10.1080/07853890.2025.2458207 39865866 PMC11774153

[98] Poto R Shamji M Marone G Durham SR Scadding GW Varricchi G . Neutrophil extracellular traps in asthma: Friends or foes? Cells. (2022) 11:3521. doi: 10.3390/cells11213521 36359917 PMC9654069

[99] Yousefi S Gold JA Andina N Lee JJ Kelly AM Kozlowski E . Catapult-like release of mitochondrial DNA by eosinophils contributes to antibacterial defense. Nat Med. (2008) 14:949–53. doi: 10.1038/nm.1855 18690244

[100] Lu Y Huang Y Li J Huang J Zhang L Feng J . Eosinophil extracellular traps drive asthma progression through neuro-immune signals. Nat Cell Biol. (2021) 23:1060–72. doi: 10.1038/s41556-021-00762-2 34616019

[101] Silveira JS Antunes GL Kaiber DB da Costa MS Ferreira FS Marques EP . Autophagy induces eosinophil extracellular traps formation and allergic airway inflammation in a murine asthma model. J Cell Physiol. (2020) 235:267–80. doi: 10.1002/jcp.28966 31206674

[102] Germic N Fettrelet T Stojkov D Hosseini A Horn MP Karaulov A . The release kinetics of eosinophil peroxidase and mitochondrial DNA is different in association with eosinophil extracellular trap formation. Cells. (2021) 10:306. doi: 10.3390/cells10020306 33546138 PMC7913244

[103] Shen K Zhang M Zhao R Li Y Li C Hou X . Eosinophil extracellular traps in asthma: Implications for pathogenesis and therapy. Respir Res. (2023) 24:231. doi: 10.1186/s12931-023-02504-4 37752512 PMC10523707

[104] Fukuchi M Miyabe Y Furutani C Saga T Moritoki Y Yamada T . How to detect eosinophil ETosis (EETosis) and extracellular traps. Allergol Int. (2021) 70:19–29. doi: 10.1016/j.alit.2020.10.002 33189567 PMC9333458

[105] Koranteng J Chung KF Michaeloudes C Bhavsar P . The role of mitochondria in eosinophil function: Implications for severe asthma pathogenesis. Front Cell Dev Biol. (2024) 12:1360079. doi: 10.3389/fcell.2024.1360079 38495619 PMC10940389

[106] Chandrasekaran R Bruno SR Mark ZF Walzer J Caffry S Gold C . Mitoquinone mesylate attenuates pathological features of lean and obese allergic asthma in mice. Am J Physiol Lung Cell Mol Physiol. (2023) 324:L141–53. doi: 10.1152/ajplung.00249.2022 36511516 PMC9902225

[107] Savin IA Zenkova MA Sen’kova AV . Bronchial asthma, airway remodeling and lung fibrosis as successive steps of one process. Int J Mol Sci. (2023) 24:16042. doi: 10.3390/ijms242216042 38003234 PMC10671561

[108] Han Y Ji X Rao J Zhang Y Chen X Gong F . TNFAIP3, PADI4, and CXCR4 as biomarkers of neutrophil extracellular traps in severe asthma. Int Immunopharmacol. (2026) 170:116118. doi: 10.1016/j.intimp.2025.116118 41475137

[109] Liu S Lin Z Zhou J Youse L Yang Q Li T . Multiomics and machine learning reveal distinct immune-metabolic signatures and diagnostic biomarkers for asthma inflammatory endotypes. ACS Omega. (2025) 10:56006–24. doi: 10.1021/acsomega.5c07657 41322535 PMC12658698

[110] Liao S-X Wang Y-W Shi L-M Zhang L-Y Zhou J Sun P-P . NCX1 reverse mode promotes calcium-dependent neutrophil extracellular trap formation and lung damage in chronic obstructive pulmonary disease. Nat Commun. (2026) 17:3801. doi: 10.1038/s41467-026-69636-1 41813659 PMC13111594

[111] Cai C Guan L Wang C Hu R Ou L Jiang Q . The role of fibroblast-neutrophil crosstalk in the pathogenesis of inflammatory diseases: A multi-tissue perspective. Front Immunol. (2025) 16:1588667. doi: 10.3389/fimmu.2025.1588667 40486514 PMC12140995

[112] Ganesh K Gomes SM Vasishta S Poojary G Thimmappa PY Peesapati S . Immuno-metabolic dysregulation in type 2 diabetes is associated with altered neutrophil functional plasticity, mitochondrial dysfunction, and compromised responses in sepsis. J Transl Med. (2026) 24:156. doi: 10.1186/s12967-026-07696-z 41520116 PMC12882159

[113] Li L Ma Y Hu Y Wang P Han S Zhang X . Site-specific inhibition of neutrophilic inflammation by low-dose nanotherapy for immunoregulatory treatment of asthma. Nano Today. (2023) 52:101957. doi: 10.1016/j.nantod.2023.101957 42574925

[114] Modak M Rowlands WM Sleiman J Attaway AH Bleecker ER Zein J . Hospitalization outcomes of patients with asthma, COPD, and asthma-COPD overlap syndrome. Chronic Obstr Pulm Dis. (2025) 12:260–73. doi: 10.15326/jcopdf.2024.0566 40554792 PMC12429536

[115] Aoshiba K Nagai A . Differences in airway remodeling between asthma and chronic obstructive pulmonary disease. Clin Rev Allergy Immunol. (2004) 27:35–43. doi: 10.1385/CRIAI:27:1:035 15347849

[116] Fuchs HJ Borowitz DS Christiansen DH Morris EM Nash ML Ramsey BW . Effect of aerosolized recombinant human DNase on exacerbations of respiratory symptoms and on pulmonary function in patients with cystic fibrosis. The pulmozyme study group. N Engl J Med. (1994) 331:637–42. doi: 10.1056/NEJM199409083311003 7503821

[117] Ortega HG Liu MC Pavord ID Brusselle GG FitzGerald JM Chetta A . Mepolizumab treatment in patients with severe eosinophilic asthma. N Engl J Med. (2014) 371:1198–207. doi: 10.1056/NEJMoa1403290 25199059

[118] Bel EH Wenzel SE Thompson PJ Prazma CM Keene ON Yancey SW . Oral glucocorticoid-sparing effect of mepolizumab in eosinophilic asthma. N Engl J Med. (2014) 371:1189–97. doi: 10.1056/NEJMoa1403291 25199060

[119] FitzGerald JM Bleecker ER Nair P Korn S Ohta K Lommatzsch M . Benralizumab, an anti-interleukin-5 receptor α monoclonal antibody, as add-on treatment for patients with severe, uncontrolled, eosinophilic asthma (CALIMA): A randomised, double-blind, placebo-controlled phase 3 trial. Lancet. (2016) 388:2128–41. doi: 10.1016/S0140-6736(16)31322-8 27609406

[120] Ramakrishnan S Russell REK Mahmood HR Krassowska K Melhorn J Mwasuku C . Treating eosinophilic exacerbations of asthma and COPD with benralizumab (ABRA): A double-blind, double-dummy, active placebo-controlled randomised trial. Lancet Respir Med. (2025) 13:59–68. doi: 10.1016/S2213-2600(24)00299-6 39615502

[121] Menzies-Gow A Corren J Bourdin A Chupp G Israel E Wechsler ME . Tezepelumab in adults and adolescents with severe, uncontrolled asthma. N Engl J Med. (2021) 384:1800–9. doi: 10.1056/NEJMoa2034975 33979488

[122] Rabe KF Nair P Brusselle G Maspero JF Castro M Sher L . Efficacy and safety of dupilumab in glucocorticoid-dependent severe asthma. N Engl J Med. (2018) 378:2475–85. doi: 10.1056/NEJMoa1804093 29782224

[123] Murphy MP Smith RAJ . Targeting antioxidants to mitochondria by conjugation to lipophilic cations. Annu Rev Pharmacol Toxicol. (2007) 47:629–56. doi: 10.1146/annurev.pharmtox.47.120505.105110 17014364

[124] Fairley LH Das S Dharwal V Amorim N Hegarty KJ Wadhwa R . Mitochondria-targeted antioxidants as a therapeutic strategy for chronic obstructive pulmonary disease. Antioxidants (Basel). (2023) 12:973. doi: 10.3390/antiox12040973 37107348 PMC10135688

[125] Agrawal A Mabalirajan U . Rejuvenating cellular respiration for optimizing respiratory function: Targeting mitochondria. Am J Physiol Lung Cell Mol Physiol. (2016) 310:L103–113. doi: 10.1152/ajplung.00320.2015 26566906

[126] Caldeira DA Weiss DJ Rocco PRM Silva PL Cruz FF . Mitochondria in focus: From function to therapeutic strategies in chronic lung diseases. Front Immunol. (2021) 12:782074. doi: 10.3389/fimmu.2021.782074 34887870 PMC8649841

[127] Barnes PJ . Oxidative stress-based therapeutics in COPD. Redox Biol. (2020) 33:101544. doi: 10.1016/j.redox.2020.101544 32336666 PMC7251237

[128] Cen M Ouyang W Zhang W Yang L Lin X Dai M . MitoQ protects against hyperpermeability of endothelium barrier in acute lung injury via a Nrf2-dependent mechanism. Redox Biol. (2021) 41:101936. doi: 10.1016/j.redox.2021.101936 33752110 PMC8005834

[129] Chen L Qin Y Liu B Gao M Li A Li X . PGC-1α-mediated mitochondrial quality control: Molecular mechanisms and implications for heart failure. Front Cell Dev Biol. (2022) 10:871357. doi: 10.3389/fcell.2022.871357 35721484 PMC9199988

[130] Lazaar AL Miller BE Donald AC Keeley T Ambery C Russell J . CXCR2 antagonist for patients with chronic obstructive pulmonary disease with chronic mucus hypersecretion: A phase 2b trial. Respir Res. (2020) 21:149. doi: 10.1186/s12931-020-01401-4 32532258 PMC7291447

[131] Keir HR Richardson H Fillmore C Shoemark A Lazaar AL Miller BE . CXCL-8-dependent and -independent neutrophil activation in COPD: Experiences from a pilot study of the CXCR2 antagonist danirixin. ERJ Open Res. (2020) 6:00583–2020. doi: 10.1183/23120541.00583-2020 33263062 PMC7682717

[132] Liu G Philp AM Corte T Travis MA Schilter H Hansbro NG . Therapeutic targets in lung tissue remodelling and fibrosis. Pharmacol Ther. (2021) 225:107839. doi: 10.1016/j.pharmthera.2021.107839 33774068

[133] Pilsczek FH Salina D Poon KKH Fahey C Yipp BG Sibley CD . A novel mechanism of rapid nuclear neutrophil extracellular trap formation in response to staphylococcus aureus. J Immunol. (2010) 185:7413–25. doi: 10.4049/jimmunol.1000675 21098229

[134] Yipp BG Kubes P . NETosis: How vital is it? Blood. (2013) 122:2784–94. doi: 10.1182/blood-2013-04-457671 24009232

[135] Wigerblad G Cao Q Brooks S Naz F Gadkari M Jiang K . Single-cell analysis reveals the range of transcriptional states of circulating human neutrophils. J Immunol. (2022) 209:772–82. doi: 10.4049/jimmunol.2200154 35858733 PMC9712146

[136] Kang Z-Y Huang Q-Y Zhen N-X Xuan N-X Zhou Q-C Zhao J . Heterogeneity of immune cells and their communications unveiled by transcriptome profiling in acute inflammatory lung injury. Front Immunol. (2024) 15:1382449. doi: 10.3389/fimmu.2024.1382449 38745657 PMC11092984

[137] Ocana A Pandiella A Privat C Bravo I Luengo-Oroz M Amir E . Integrating artificial intelligence in drug discovery and early drug development: A transformative approach. biomark Res. (2025) 13:45. doi: 10.1186/s40364-025-00758-2 40087789 PMC11909971

[138] Chen H Shi Y Yuan M Li H Liu X . Integrating multi-omics and artificial intelligence fuels advanced target identification and drug discovery. Biotechnol Adv. (2026) 87:108785. doi: 10.1016/j.bioteChadv.2025.108785 41461245

[139] Choi H Yoo H Lee JY Park J Jeon K . Plasma mitochondrial DNA and necroptosis as prognostic indicators in critically ill patients with sepsis. Biomedicines. (2022) 10:2386. doi: 10.3390/biomedicines10102386 36289650 PMC9598411

[140] Sin DD . What single cell RNA sequencing has taught us about chronic obstructive pulmonary disease. Tuberc Respir Dis (Seoul). (2024) 87:252–60. doi: 10.4046/trd.2024.0001 38369875 PMC11222093

[141] Yu J Xiao T Pan Y He Y Tan J . Single cell transcriptomics genomics based on machine learning algorithm: Constructing and validating neutrophil extracellular trap gene model in COPD. Int J Gen Med. (2025) 18:2247–61. doi: 10.2147/IJGM.S516139 40308227 PMC12042204

[142] Lefrançais E Mallavia B Zhuo H Calfee CS Looney MR . Maladaptive role of neutrophil extracellular traps in pathogen-induced lung injury. JCI Insight. (2018) 3:e98178, 98178. doi: 10.1172/jci.insight.98178 29415887 PMC5821185

[143] Santos Bonilha C Protasio Veras F . Mapping benefit, risk, and opportunity in PAD4 inhibition. Front Immunol. (2026) 17:1769421. doi: 10.3389/fimmu.2026.1769421 41836413 PMC12979119

[144] Schoen J Euler M Schauer C Schett G Herrmann M Knopf J . Neutrophils’ extracellular trap mechanisms: From physiology to pathology. Int J Mol Sci. (2022) 23:12855. doi: 10.3390/ijms232112855 36361646 PMC9653572

[145] Barnes PJ . Corticosteroid resistance in patients with asthma and chronic obstructive pulmonary disease. J Allergy Clin Immunol. (2013) 131:636–45. doi: 10.1016/j.jaci.2012.12.1564 23360759

[146] Wadhwa R Dua K Adcock IM Horvat JC Kim RY Hansbro PM . Cellular mechanisms underlying steroid-resistant asthma. Eur Respir Rev. (2019) 28:190096. doi: 10.1183/16000617.0096-2019 31636089 PMC9488801

[147] Li L-B Leung DYM Martin RJ Goleva E . Inhibition of histone deacetylase 2 expression by elevated glucocorticoid receptor beta in steroid-resistant asthma. Am J Respir Crit Care Med. (2010) 182:877–83. doi: 10.1164/rccm.201001-0015OC 20538962 PMC2970859

[148] Burczyk G Kolaczkowska E . Fumarate inhibits the formation of neutrophil extracellular traps (NETs) in a Nrf2-controlled and annexin-A1-dependent manner associated with mitochondrial fusion. Front Immunol. (2026) 17:1770063. doi: 10.3389/fimmu.2026.1770063 41988179 PMC13076181

[149] Castellani S D’Oria S Diana A Polizzi AM Di Gioia S Mariggiò MA . G-CSF and GM-CSF modify neutrophil functions at concentrations found in cystic fibrosis. Sci Rep. (2019) 9:12937. doi: 10.1038/s41598-019-49419-z 31506515 PMC6736848

[150] Esteve-Codina A Hofer TP Burggraf D Heiss-Neumann MS Gesierich W Boland A . Gender specific airway gene expression in COPD sub-phenotypes supports a role of mitochondria and of different types of leukocytes. Sci Rep. (2021) 11:12848. doi: 10.1038/s41598-021-91742-x 34145303 PMC8213687

[151] Mariotti B Gasperini S Bracaglia C Frigenti C Sartori G di Chiara C . Sex-dependent transcriptional and epigenetic regulation of neutrophil inflammatory programs in COPD. Front Immunol. (2026) 17:1824596. doi: 10.3389/fimmu.2026.1824596 42282958 PMC13252782

[152] Che J Najer A Blakney AK McKay PF Bellahcene M Winter CW . Neutrophils enable local and non-invasive liposome delivery to inflamed skeletal muscle and ischemic heart. Adv Mater. (2020) 32:e2003598. doi: 10.1002/adma.202003598 33103807 PMC7613371

[153] Zhao Y Zhang H Zhang Q Tao H . Research progress of neutrophil-mediated drug delivery strategies for inflammation-related disease. Pharmaceutics. (2023) 15:1881. doi: 10.3390/pharmaceutics15071881 37514067 PMC10384340

[154] Liu X Liu Y Guo W Li X Han Y Huo Q . Targeting NETosis in the lung: Respiratory delivery of neutrophil-specific NEBP-liposomes for enhanced therapy of acute lung injury. Mol Pharm. (2026) 23:3666–74. doi: 10.1021/acs.molpharmaceut.6c00094 42241382 PMC13344298

